# Reconstruction of human protein interolog network using evolutionary conserved network

**DOI:** 10.1186/1471-2105-8-152

**Published:** 2007-05-10

**Authors:** Tao-Wei Huang, Chung-Yen Lin, Cheng-Yan Kao

**Affiliations:** 1 Department of Computer Science and Information Engineering, National Taiwan University, Taipei 106, Taiwan; 2 Institute of Information Science, Academia Sinica, Taipei 115, Taiwan; 3 Division of Biostatistics and Bioinformatics, National Health Research Institutes, Taipei 115, Taiwan; 4 Institute of Fishery Science, National Taiwan University, Taipei 106, Taiwan; 5 Institute for Information Industry, Taipei 106, Taiwan

## Abstract

**Background:**

The recent increase in the use of high-throughput two-hybrid analysis has generated large quantities of data on protein interactions. Specifically, the availability of information about experimental protein-protein interactions and other protein features on the Internet enables human protein-protein interactions to be computationally predicted from co-evolution events (interolog). This study also considers other protein interaction features, including sub-cellular localization, tissue-specificity, the cell-cycle stage and domain-domain combination. Computational methods need to be developed to integrate these heterogeneous biological data to facilitate the maximum accuracy of the human protein interaction prediction.

**Results:**

This study proposes a relative conservation score by finding maximal quasi-cliques in protein interaction networks, and considering other interaction features to formulate a scoring method. The scoring method can be adopted to discover which protein pairs are the most likely to interact among multiple protein pairs. The predicted human protein-protein interactions associated with confidence scores are derived from six eukaryotic organisms – rat, mouse, fly, worm, thale cress and baker's yeast.

**Conclusion:**

Evaluation results of the proposed method using functional keyword and Gene Ontology (GO) annotations indicate that some confidence is justified in the accuracy of the predicted interactions. Comparisons among existing methods also reveal that the proposed method predicts human protein-protein interactions more accurately than other interolog-based methods.

## Background

Large-scale protein-protein interactions (PPIs) have been experimentally identified in several eukaryotic model organisms, such as *Drosophila melanogaster *[1-3], *Caenorhabditis elegans *[4,5], and *Saccharomyces cerevisiae *[6-9]. Moreover, thousands of PPIs have been collected from web databases including BIND [10], CYGD [11], DIP [12], BioGRID [13], IntAct [14], and MINT [15]. Although the mammalian interactions, MPPI [16], have been published, the amount of the data with similar scale has not been described. The large-scale set of interactions of human proteins is still hard to determine directly.

Many computational methods have been developed to predict protein-protein interactions. A phylogenetic profile method [17] describes the presence or absence of proteins among different organisms with sequenced genomes. Proteins have similar phylogenetic profiles, between which functional links can be detected. The gene or domain fusion method [18,19] describes a pair of proteins encoded as separate genes in one organism and fused into a single protein in another organism. Such a pair of proteins can be inferred by the function link, particularly among metabolic pathways. In the gene neighbor or gene order method [20-22], the genes that encode two proteins are adjacent in chromosome proximity in several organisms, and are likely to be functionally linked. However, this method exploits the prevalance of operons in prokaryotes, but operons appear to be uncommon in eukaryoyes such as humans. Predictions using interologs [5] are based on the theory that proteins interacting in one organism co-evolve such that their respective orthologs maintain the ability to interact in another organism. The interolog concept has been applied to predict human protein interactions [23-29]. Some bioinformatics models [30,31] have also been developed to detect interactions among proteins by probability and machine-learning methods and the literature text-mining approach [32-34] based on natural language processing. Bader *et al*. developed a logistic regression approach [35] that adopts employs statistical and topological descriptors to predict the biological relevance of PPIs obtained from high-throughput screening for yeast. Other sources of information, such as mRNA expression, genetic interactions and database annotations, are subsequently used to validate the model predictions. Lu *et al*. used a simple *Naive Bayes *classifier to integrate diverse sources of genomic evidence, ranging from co-expression relationships to phylogenetic profiling similarity [36].

The greatest challenge in predicting human PPIs using the interolog-based method is that the high-throughput interactions generate too many false positives when applied to phylogenetically distant organisms or lower eukaryotes [37], and some researchers have suggested that only 50% of yeast two-hybrid interactions are reliable [38]. Therefore, other filtering examinations of features and scoring schema should be further considered in order to increase the confidence in the prediction of human interactions performed by the interolog-based method. This study constructs human PPI maps from six eukaryotes, namely rat, mouse, fly, worm, thale cress and baker's yeast. The quasi-clique is analyzed and determined as a relative conservation score from the protein interaction networks in each organism. The other feature scores further drawn from spatial proximity (sub-cellular localization and tissue-specificity), temporal synchronicity (cell-cycle stage) and domain-domain combinations are also inspected, to obtain human PPI networks with confidence scores.

## Results and discussion

### Predicted human protein interactions

All protein access codes, such as NCBI GI number or RefSeq ID, were converted into non-redundant UniProt IDs. Table 1 shows the non-redundant (nr) total set of the originally predicted human protein-protein interactions (interologs) derived from six reference organisms. One-to-many mappings exist across species in the InParanoid-predicted data set, and are applied to identify protein orthologs. The total data set of 90, 871 human PPIs was obtained by the proposed method without cutoff by confidence score (*CS*). A total of 90, 871 protein interactions were predicted (see Additional File 1).

**Table 1:** Number and sources of predicted interactions inferred from each reference organism.

	Reference organisms						
		
	Rat	Mouse	Fly	Worm	Thale cress	Baker's yeast	Total (nr)
Proteins	1,183	2,962	9,910	3,607	551	6,590	24,803
Interactions	1,344	3,895	44,119	7,690	2,134	115,903	175,085
Predicted interologs (nr)	476	1,212	13,131	8,429	1,384	82,425	90,871

**Figure 1:**
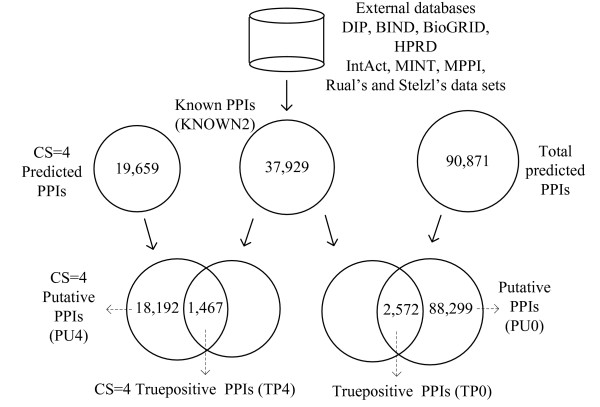
**Schematic illustration of interaction data**. Schematic illustration of sets of known (KNOWN2), predicted true positive (TP0) and predicted putative (PU0) interaction data. The confidence score (*CS *= 4 herein) can be used to identify interaction sets (TP4 and PU4) quantitatively and filter out the predicted interactions with lower confidence.

The known human interactions (indicated as KNOWN) were downloaded from external databases BIND, BioGRID, DIP, HPRD, IntAct, MINT and MPPI. The KNOWN2 data set was derived from KNOWN with the addition of two recently published experimental data sets of human PPIs [39,40]. The proposed method predicted all 2, 572(2.83%) true positive (TP0) and 88, 299(97.17%) putatives (PU0) interaction data sets when applying the threshold *CS *≥ 0. A threshold of *CS *≥ 4 achieved 1, 467(7.46%) true positives (TP4) and 18, 192(92.53%) putatives (PU4). Figure 1 summarizes the results that showing the relationship among these data sets. The following evaluation compares the functional annotations among the KNOWN2, TP4, TP0, PU4, PU0, and random interaction data sets (RANDOMS).

### Evaluation

The experimental human PPIs and standard benchmark are limited from well-known databases and few interactions are known completely. Therefore, the absence of interactions between proteins from the experimental databases does not indicate that the interactions are negative. Given this limited knowledge, functional keyword annotation and GO term matching were tested to determine the accuracy of measurement of various interaction data sets.

#### Testing for true positives

Table 2 presents the successfully predicted human PPIs (true positives) from different reference organisms in the first evaluation. The accuracy of combining the predicted human interactions from various reference organisms was found to exceed that of a single reference organism. Although the large-scale and protein interactions of rat and mouse have not yet been completed, these two mammal model organisms can be used to identify higher proportion of predicted true positives, 317476=66.60%
 MathType@MTEF@5@5@+=feaafiart1ev1aaatCvAUfKttLearuWrP9MDH5MBPbIqV92AaeXatLxBI9gBaebbnrfifHhDYfgasaacH8akY=wiFfYdH8Gipec8Eeeu0xXdbba9frFj0=OqFfea0dXdd9vqai=hGuQ8kuc9pgc9s8qqaq=dirpe0xb9q8qiLsFr0=vr0=vr0dc8meaabaqaciaacaGaaeqabaqabeGadaaakeaadaWcaaqaaiabiodaZiabigdaXiabiEda3aqaaiabisda0iabiEda3iabiAda2aaacqGH9aqpcqaI2aGncqaI2aGncqGGUaGlcqaI2aGncqaIWaamcqGGLaqjaaa@3920@ and 4741212=39.11%
 MathType@MTEF@5@5@+=feaafiart1ev1aaatCvAUfKttLearuWrP9MDH5MBPbIqV92AaeXatLxBI9gBaebbnrfifHhDYfgasaacH8akY=wiFfYdH8Gipec8Eeeu0xXdbba9frFj0=OqFfea0dXdd9vqai=hGuQ8kuc9pgc9s8qqaq=dirpe0xb9q8qiLsFr0=vr0=vr0dc8meaabaqaciaacaGaaeqabaqabeGadaaakeaadaWcaaqaaiabisda0iabiEda3iabisda0aqaaiabigdaXiabikdaYiabigdaXiabikdaYaaacqGH9aqpcqaIZaWmcqaI5aqocqGGUaGlcqaIXaqmcqaIXaqmcqGGLaqjaaa@39F8@, respectively (Table 1 and Table 2). Therefore, human interactions can be confidently predicted from multiple mammalian organisms and higher eukaryotes.

**Table 2:** Number of human interactions (true positives) successfully predicted from each reference organism in different experimental databases.

		Predicted true positive interactions						
			
Databases	Human	Rat	Mouse	Fly	Worm	Thale cress	Baker's yeast	Total (nr)
BIND	1,755	19	84	45	38	5	191	327
BioGRID	15,578	81	327	212	133	37	894	1516
DIP	703	9	50	23	11	2	77	150
HPRD	18,767	303	415	233	168	35	938	1,912
IntAct	7,046	18	95	93	51	11	523	709
MINT	3,236	16	79	73	49	17	305	478
MPPI	247	3	21	10	7	4	43	77
Rual	4,044	9	38	46	32	4	223	307
Stelzl	2,889	9	23	29	23	2	184	238

Total (nr)	37,929	317	474	335	212	45	1,433	2,572

#### Testing scoring method

**Figure 2:**
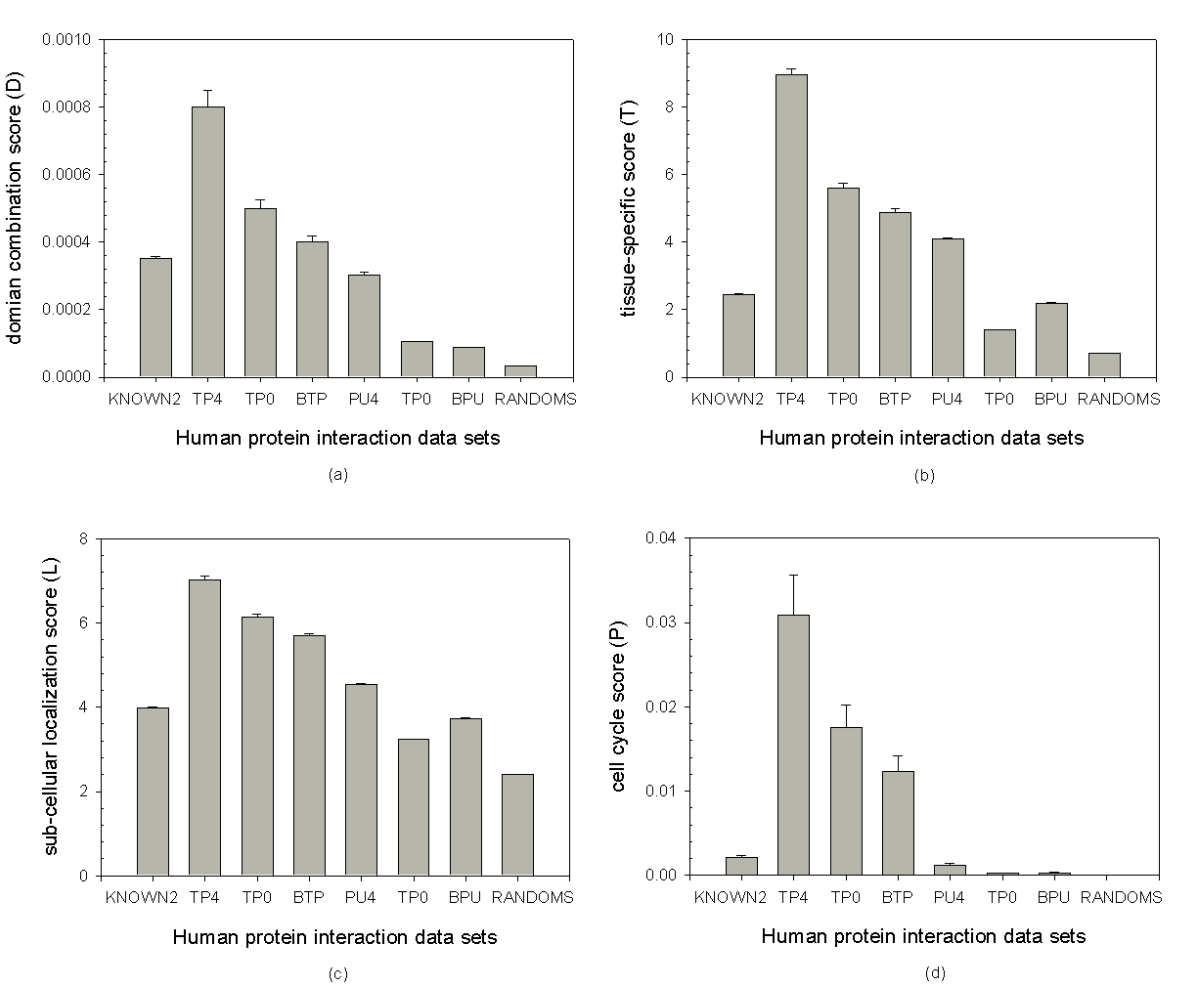
**Each feature score for all data sets**. Each feature score for all data sets; x-axis is the feature type, and y-axis is the corresponding raw feature score (mean value). The predicted data sets with confidence score (*CS *= 4) (TP4 and PU4) have similar or higher feature scores than the known interaction data sets with two recently published experimental data sets (KNOWN2), data sets for true positive and putative predicted from BLAST mapping method (BTP and BPU) and randomly generated data sets (RANDOMS).

Each feature score of each data set was evaluated to determine whether the proposed scoring method was associated with more accurate predictions of interactions. The data sets predicted by BLAST search method (BTP and BPU are data sets for true positive and putative, respectively) were also compared with our predicted data sets. In Figure 2, each feature score was the original raw score without normalization, revealing that the data sets (TP4 and PU4) predicted by our approach have similar but higher feature scores than those of the known interaction data sets (KNOWN2) and the randomly generated data sets (RANDOMS). The distributions of the various components of the confidence metrics and ANOVA tests between these interaction data sets were listed (see Additional File 2). The differences between these data sets are statistically significant.

#### Testing functional annotation

Interacting proteins commonly have similar functions. Additionally, researchers should be able to validate the functions of predicted protein pairs. The interactions predicted by the proposed method were optimized in terms of UniProt functional keyword annotations, GO 'molecular function' (MF) and GO 'biological process' (BP). Their relevant GO terms such as 'molecular function unknown', 'obsolete molecular function', 'biological process unknown' and 'obsolete biological process' were discarded.

Equations (1), (2), and (3) define the Jaccard coefficient of the UniProt keyword, and the deepest depth of common ancestor GO terms in MF and BP categories, *UK*, *GMF *and *GBP*, respectively.

UK=KaT∗KbKaT∗Ka+KbT∗Kb−KaT∗Kb
 MathType@MTEF@5@5@+=feaafiart1ev1aaatCvAUfKttLearuWrP9MDH5MBPbIqV92AaeXatLxBI9gBaebbnrfifHhDYfgasaacH8akY=wiFfYdH8Gipec8Eeeu0xXdbba9frFj0=OqFfea0dXdd9vqai=hGuQ8kuc9pgc9s8qqaq=dirpe0xb9q8qiLsFr0=vr0=vr0dc8meaabaqaciaacaGaaeqabaqabeGadaaakeaacqWGvbqvcqWGlbWscqGH9aqpdaWcaaqaaiabdUealnaaDaaaleaacqWGHbqyaeaacqWGubavaaGccqGHxiIkcqWGlbWsdaWgaaWcbaGaemOyaigabeaaaOqaaiabdUealnaaDaaaleaacqWGHbqyaeaacqWGubavaaGccqGHxiIkcqWGlbWsdaWgaaWcbaGaemyyaegabeaakiabgUcaRiabdUealnaaDaaaleaacqWGIbGyaeaacqWGubavaaGccqGHxiIkcqWGlbWsdaWgaaWcbaGaemOyaigabeaakiabgkHiTiabdUealnaaDaaaleaacqWGHbqyaeaacqWGubavaaGccqGHxiIkcqWGlbWsdaWgaaWcbaGaemOyaigabeaaaaaaaa@4F65@

GMF=∑i=1i∗(% of PPI share ancestor GO term at depth i in MF)
 MathType@MTEF@5@5@+=feaafiart1ev1aaatCvAUfKttLearuWrP9MDH5MBPbIqV92AaeXatLxBI9gBaebbnrfifHhDYfgasaacH8akY=wiFfYdH8Gipec8Eeeu0xXdbba9frFj0=OqFfea0dXdd9vqai=hGuQ8kuc9pgc9s8qqaq=dirpe0xb9q8qiLsFr0=vr0=vr0dc8meaabaqaciaacaGaaeqabaqabeGadaaakeaacqWGhbWrcqWGnbqtcqWGgbGrcqGH9aqpdaaeqbqaaiabdMgaPjabgEHiQiabcIcaOiabcwcaLiabbccaGiabb+gaVjabbAgaMjabbccaGiabbcfaqjabbcfaqjabbMeajjabbccaGiabbohaZjabbIgaOjabbggaHjabbkhaYjabbwgaLjabbccaGiabbggaHjabb6gaUjabbogaJjabbwgaLjabbohaZjabbsha0jabb+gaVjabbkhaYjabbccaGiabbEeahjabb+eapjabbccaGiabbsha0jabbwgaLjabbkhaYjabb2gaTjabbccaGiabbggaHjabbsha0jabbccaGiabbsgaKjabbwgaLjabbchaWjabbsha0jabbIgaOjabbccaGiabdMgaPjabbccaGiabbMgaPjabb6gaUjabbccaGiabb2eanjabbAeagjabbMcaPaWcbaGaemyAaKMaeyypa0JaeGymaedabeqdcqGHris5aaaa@7343@

GBP=∑i=1i∗(% of PPI share ancestor GO term at depth i in BP)
 MathType@MTEF@5@5@+=feaafiart1ev1aaatCvAUfKttLearuWrP9MDH5MBPbIqV92AaeXatLxBI9gBaebbnrfifHhDYfgasaacH8akY=wiFfYdH8Gipec8Eeeu0xXdbba9frFj0=OqFfea0dXdd9vqai=hGuQ8kuc9pgc9s8qqaq=dirpe0xb9q8qiLsFr0=vr0=vr0dc8meaabaqaciaacaGaaeqabaqabeGadaaakeaacqWGhbWrcqWGcbGqcqWGqbaucqGH9aqpdaaeqbqaaiabdMgaPjabgEHiQiabcIcaOiabcwcaLiabbccaGiabb+gaVjabbAgaMjabbccaGiabbcfaqjabbcfaqjabbMeajjabbccaGiabbohaZjabbIgaOjabbggaHjabbkhaYjabbwgaLjabbccaGiabbggaHjabb6gaUjabbogaJjabbwgaLjabbohaZjabbsha0jabb+gaVjabbkhaYjabbccaGiabbEeahjabb+eapjabbccaGiabbsha0jabbwgaLjabbkhaYjabb2gaTjabbccaGiabbggaHjabbsha0jabbccaGiabbsgaKjabbwgaLjabbchaWjabbsha0jabbIgaOjabbccaGiabdMgaPjabbccaGiabbMgaPjabb6gaUjabbccaGiabbkeacjabbcfaqjabbMcaPaWcbaGaemyAaKMaeyypa0JaeGymaedabeqdcqGHris5aaaa@733F@

where *K*_*a*_, *K*_*b *_are the keyword vectors of interacting protein pairs a and b, respectively. For example, in *K*_*a *_= [1, 0, 1, 0, 1], the presence or absence of a keyword are represented as 1 or 0, respectively. Protein self-interactions or homo-dimers tend to have high scores, and always share the same functional annotations. Hence, these interactions were eliminated from the predicted pairs to eliminate bias in the results.

**Figure 3:**
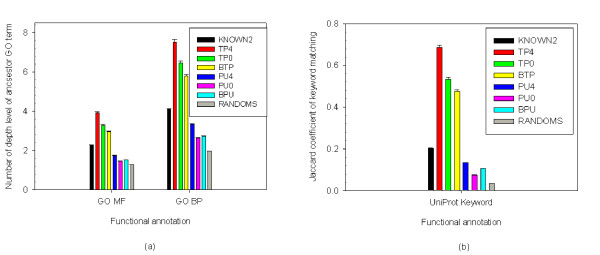
**Testing of functional annotation**. Testing of functional annotation between all data sets. (a) Mean depth level of common ancestor GO term in 'molecular function' (MF) or 'biological process' (BP) categories. (b) Mean of Jaccard coefficient of UniProt keyword matching.

First, the number of interaction pairs sharing at least one UniProt overlapping functional keyword was determined to verify the accuracy of the predicted interactions. Second, the number of interaction pairs sharing common GO annotations at a particular depth in the GO 'molecular function' and 'biological process' hierarchy was analyzed to confirm that the results and that were not just a general GO term applied. Comparisons were made among KNOWN2, TP4, TP0, BTP, PU4, PU0, BPU and RANDOMS data sets (Figure 3).

Finally, the probability that two proteins share the same UniProt functional keyword by chance is determined through the hypergeometric distribution [41]. The *p*-value is obtained by the following equation:

p=∑xn(Mx)(N−Mn−x)(Nn)
 MathType@MTEF@5@5@+=feaafiart1ev1aaatCvAUfKttLearuWrP9MDH5MBPbIqV92AaeXatLxBI9gBaebbnrfifHhDYfgasaacH8akY=wiFfYdH8Gipec8Eeeu0xXdbba9frFj0=OqFfea0dXdd9vqai=hGuQ8kuc9pgc9s8qqaq=dirpe0xb9q8qiLsFr0=vr0=vr0dc8meaabaqaciaacaGaaeqabaqabeGadaaakeaacqWGWbaCcqGH9aqpdaaeWbqaamaalaaabaWaaeWaaeaafaqabeGabaaabaGaemyta0eabaGaemiEaGhaaaGaayjkaiaawMcaamaabmaabaqbaeqabiqaaaqaaiabd6eaojabgkHiTiabd2eanbqaaiabd6gaUjabgkHiTiabdIha4baaaiaawIcacaGLPaaaaeaadaqadaqaauaabeqaceaaaeaacqWGobGtaeaacqWGUbGBaaaacaGLOaGaayzkaaaaaaWcbaGaemiEaGhabaGaemOBa4ganiabggHiLdaaaa@4533@

where *N *and *M *denote the total number of proteins in the population, and the number of proteins that have a particular functional keyword, respectively, and *n *and *x *denote the total number of proteins in the set, and the number of proteins annotated with the particular functional keyword, respectively. Since a pair of proteins is observed, both *n *and *x *are equal to 2. A protein pair is treated as enriched by a UniProt functional if the corrected *p*-value is ≤ 0.05. The total of 90, 871 predicted interactions with this *p*-value are listed (see Additional File 1).

#### Testing conservation score (*C*) and interolog score (*I*)

Table 3 and Table 4 show present the effectiveness of conservation (*C*) and interolog scores (*I*) based on the quasi-clique of protein networks. The raw conservation score and interolog score and corresponding standard error of true positive and putative interaction data sets from different InParanoid score (0.0 to 1.0) were evaluated. The result reveals that the conservation and interolog scores in the true positive data set were higher than those in the putative data set.

**Table 3:** Mean and standard error of Conservation score (*C*) among the different InParanoid score (*IP*) interaction data sets.

	True positives		Putative	
	
InParanoid score	Mean of C score	Std Err	Mean of C score	Std Err
*IP *> 0.0(*CS *≥ 4)	2,278.54	15.31	519.62	28.98
*IP *> 0.0(*CS *≥ 0)	725.17	4.73	335.75	17.43
*IP *≥ 0.1	762.24	5.09	341.67	17.92
*IP *≥ 0.2	812.78	5.60	344.13	18.38
*IP *≥ 0.3	853.73	5.95	353.66	18.89
*IP *≥ 0.4	896.72	6.34	359.68	19.54
*IP *≥ 0.5	958.65	6.90	365.77	20.18
*IP *≥ 0.6	953.82	7.17	367.83	20.55
*IP *≥ 0.7	984.81	7.74	361.30	20.99
*IP *≥ 0.8	979.91	8.07	355.88	21.03
*IP *≥ 0.9	992.17	8.42	352.37	21.21
*IP *= 1.0	1,005.40	8.85	352.46	21.34

**Table 4:** Mean and standard error of Interolog score (*I*) among the different InParanoid score (*IP*) interaction data sets.

	True positives		Putative	
	
InParanoid score	Mean of I score	Std Err	Mean of I score	Std Err
*IP *> 0.0(*CS *≥ 4)	506.59	3.58	120.50	6.88
*IP *> 0.0(*CS *≥ 0)	138.16	1.01	75.94	4.10
*IP *≥ 0.1	152.02	1.11	78.41	4.23
*IP *≥ 0.2	171.81	1.25	81.96	4.43
*IP *≥ 0.3	184.67	1.34	84.33	4.56
*IP *≥ 0.4	199.23	1.46	87.07	4.73
*IP *≥ 0.5	218.09	1.61	89.62	4.91
*IP *≥ 0.6	225.91	1.73	91.17	5.04
*IP *≥ 0.7	239.93	1.90	91.48	5.21
*IP *≥ 0.8	243.44	2.00	90.76	5.25
*IP *≥ 0.9	248.07	2.10	90.20	5.31
*IP *= 1.0	252.37	2.21	90.27	5.34

### Comparisons

#### Comparison with cut-off scores

Table 5 indicates that InParanoid can predict 1, 918(5.27%) and 2, 572(2.83%) true positive interologs for one-to-one mapping and one-to-many mapping, respectively. The table also shows the precision values, given by (TP/(TP+FP)) and the recall, given by (TP/(TP+FN)), where TP, FP and FN denote the numbers of true positive, false positive and false negative interactions in the predicted data sets, respectively. True positives are the overlaps between predicted positive data set and all known human interactions (KNOWN2); false negatives are the overlaps between predicted negative data set and all known human interactions (KNOWN2), and false positives are the predicted positive data sets that are absent from the true positives (i.e. the putatives in this case).

**Table 5:** Number of human interactions (true positives) predicted from BLAST with minimum E-value and InParanoid.

Data sets	Predicted interologs	True positives	Putatives	Precision	Recall
BLAST	84,501	4,130	80,371	4.89%	-*
InParanoid (1-to-1 mapping)	36,376	1,918	34,458	5.27%	-*
InParanoid (1-to-many mapping, *CS *≥ 0)	90,871	2,572	88,299	2.83%	-*
InParanoid (1-to-many mapping, *CS *≥ 4)	19,659	1,467	18,192	7.46%	57.04%

* : the predicted data set do not contain negative data to calculate false negative (FN) to obtain recall = (TP/(TP+FN))

The cut-off threshold of confidence score (*CS*), equation (8), was identified to increase the true positive ratio and indicate the relationship between the number of predicted interactions and the coverage of known interactions. The maximum precision was obtained by a threshold of *CS *≥ 4. Table 6 shows the relationship between cut-off threshold and predicted data sets from the 175, 085 known interactions in the six reference organisms.

**Table 6:** Relationship between cut-off threshold and predicted human interactions (true positives).

Cut-off threshold	Predicted interologs	True positives	Putatives	Precision	Recall
*CS *≥ 0	90,871	2,572	88,299	2.83%	-*
*CS *≥ 1	59,919	2,473	57,446	4.13%	96.15%
*CS *≥ 2	41,828	2,222	39,606	5.31%	86.39%
*CS *≥ 3	27,048	1,772	25,276	6.55%	68.90%
*CS *≥ 4	19,659	1,467	18,192	7.46%	57.04%
*CS *≥ 5	14,344	1,226	13,118	8.55%	47.67%
*CS *≥ 6	11,334	1,021	10,313	9.01%	39.70%
*CS *≥ 7	8,374	859	7,515	10.26%	33.40%

* : the predicted data set do not contain negative data to calculate false negative (FN) to obtain recall = (TP/(TP+FN))

#### Comparison with BLAST data sets

All of the 175, 085 known interactions (Table 1) from the six reference organisms were used in the orthology search by BLAST with minimum E-value (the *E *≤ 0.005 was configured in the BLAST tool). The protein sequences were downloaded from UniProt. The InParanoid one-to-one mapping (InPranoid score = 1.0) and one-to-many mappings (InPranoid score ≥ 0.0) were also compared, as were the InParanoid data sets with threshold *CS *= 4. Table 5 shows the results of these predictions. Although BLAST can more true positive interologs in quantity than the InParanoid method, it also produced a higher putative ratio. The predicted and true positive ratios reveal that InParanoid can distinguish potential true orthologs. The BTP and BPU are data sets for true positive and putative predicted from BLAST mapping method, respectively. The scoring method testing results are also presented (see Figure 2 and Additional File 2).

#### Comparison with experimental data sets

**Figure 4:**
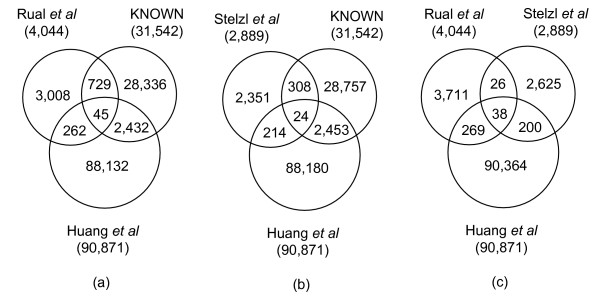
**Comparisons among experimental data sets**. Comparisons among two experimental data sets (Rual's and Stelzl's data sets), known databases (KNOWN) and our results (Huang).

All of the proteins were mapped to UniProt Entry ID, and proteins (and their interactions) that could not be confidently mapped were eliminated. Figure 4 presents the overlap among various interacting data sets, including two human experimental networks [39,40] and our predicted interlogs from six reference organisms (Huang *et al*.). Surprisingly, the results of the proposed interolog-based approach and the experimental high-throughput method did not overlap significantly, revealing that the methods applied to detect interactions have different biases. Therefore, two methods (interolog-based and experimental method) may reveal different and partial sub-networks of the whole human protein interaction network. The proposed method is based on evolutionarily conserved interologs, and can not distinguish between species-specific interactions from the two experimental data sets.

#### Comparison with interolog-based approach

The proposed method was compared with other interolog-based methods for predicting human PPIs, namely HomoMINT [28], HPID [25], IPPRED [24], the method of Lehner *et al*.'s group [27], OPHID [23], POINT [26] and Rhodes *et al*.'s method [29].

The properties of the ortholog identification methods and other features are as follows.

• An ortholog identification method indicates the orthologs between model organisms. Orthologs between organisms do not have a one-to-one relationship with BLAST search (B) or BLAST search with E-value (BE); yet one-to-many and many-to-many mappings exist. The InParanoid clustering algorithm distinguishes potential true orthologs from paralogs according to the InParanoid score (*IP*). Although similar structures typically share similar biological functions, the structural classification at the protein superfamily level (SS) is not trivial in the identification of structural similarities at the human protein level on the large scale.

• Other features indicate that some other factors affecting their interactions are considered. The quasi-clique with maximal conservation score (*C*), domain-domain combinations (*D*), sub-cellular localization (*L*), cell-cycle phase (*P*) and tissue-specificity (*T*) were also carefully examined in this study. Other existing methods apply the 'biological process' (BP) and 'molecular function' (MF) annotations in the GO hierarchy.

The brief comparisons in Table 7 reveal that the proposed method predicts results based on the relative conservation score and the other feature scores to obtain human PPI networks through confidence scores. A confidence score allows researchers to identify interactions qualitatively from objective and biologically reasonable judgement, rather than using a large quantity of interacting data without prioritized selection.

**Table 7:** Comparisons with other interolog-based approach for predicting human PPIs.

	Ortholog mapping	Other features	Predicted interologs	True positives
HomoMINT	IP	-	9,749	694
HPID	BE, SS	D, L, MF	-	-
IPPRED	BE	-	-	-
Lehner *et. al*.	IP	L	-	-
OPHID	BE	D, T, L	23,889	800
POINT	B	L, P	-	-
Rhodes *et. al*.	IP	D, T, BP	39, 816	830
Huang *et. al*.	IP, C	D, T, L, P	90, 871	2, 572

### Biological significance

Many predicted pairs have been identified in existing known human PPI databases (KNOWN) and the two human experimental PPIs data sets (as shown in Figure 4). The top 20 predictions that were not identified or not present in the existing databases were listed (see Additional File 3) using the proposed prediction system, and indicate that some top predicted protein interacting pairs were manifestations of their potentially physical interactions. For example, for the top 1 PLK1 and STK6 interaction, PLK1 (polo-likekinase1) has just been reported this year that it interacts with Aurora-B in playing critical roles in the regulation of chromosomal dynamics [42]. STK6 is also known as Aurora-A. The kinase domains of Aurora-A and Aurora-B share more than 70% of their sequence data. Most importantly, in 3D structure, they are likely to share partially similar surface features [43]. Therefore, the interaction of Aurora-A (i.e., STK6) with PLK1 (top1 interaction) is not surprising. ORC1, the origin recognition complex protein, binds specifically to origins of replication, and serves as a platform for the assembly of additional initial factors including MCM and CDC6 proteins. MCM proteins form a hexameric structure complex with 6 subunits, namely MCM2, MCM3, MCM4, MCM5, MCM6 and MCM7 [44]. To date, ORC1 been confirmed to interact with MCM2 and MCM7. ORC1 can also be reasonably expected to interact with MCM4 (top 2 interaction) and MCM6 (top 5 interaction), because they are all localized in a complex or origin recognition site. Furthermore, since MCM proteins form a hexamer, MCM5 can reasonably be expected to interact with MCM6 (top 3 interaction), and MCM5 can be expected to interact with MCM4 (top 4 interaction). These findings reveal that constructing a protein-protein interaction network allows novel interacting proteins to be identified. All proteins of the prediction pairs are linked to a human disease in the OMIM database [45] whenever possible (see Additional File 3). Therefore, the interaction network can be further extended through these annotated disease-associated proteins. Moreover, these predicted interactions have high conservation (*C*) and interolog (*I*) scores (Table 3 and Table 4, respectively), revealing that these interactions are evolutionarily conserved across species.

### Discussion

Important high-throughput approaches such as yeast two-hybrid have recently been applied to systematically identify PPIs in humans (Figure 4). Surprisingly, the experimental results of the proposed and high-throughput methods did not overlap significantly, indicating that different biases exist because of the approaches applied to detect interactions. Hence, two methods (interolog-based and experimental methods) may indicate different and partial sub networks of the complete human-protein interaction network.

The accuracy of the predicted interactions depends mainly on the quality and completeness of the reference model organism interaction data sets. Although only a subset of the known interactions in the human interaction network can currently be accurately predicted (Table 2), the accuracy can be improved by large-scale protein interaction data in 'higher' eukaryotic reference model organisms in the future. The orthologous relationship between sequence and function is difficult to evaluate, because no clear measurement of functional similarity between any pair of proteins is made. Many one-to-many and many-to-many mappings exist across species, and can be used to identify protein orthologs. The InParanoid algorithm was applied because several proteins from so-called 'lower' eukaryotes have many co-orthologs in humans, and can be identified using InParanoid, but not with a simple one-to-one sequence similarity search based on BLAST or structural classification at the protein superfamily level.

The Interolog [5] concept was previously proposed to predict *C. elegans *PPIs from yeast. This study presents 'Interolog' as a concrete method for predicting human PPIs from those of six 'lower' eukaryotes. However, high-throughput interactions with false positives and false negatives have been noted in some eukaryotes [37]. This study utilized other features and scoring schema to derive the confidence with which human interactions are predicted using the interolog-based method. Computational analysis can be applied to determine conservation scores and other feature scores, and is readily extensible to any newly sequenced genomes. Users can construct many genome-wide PPI networks with high confidence using interolog mapping and the proposed scoring method. This concept can also be applied to discover transcription networks, such as simultaneous protein-DNA and protein-protein interaction networks [46].

## Conclusion

The evolution of PPIs from the relative conservation score is comprehensively assessed by finding a quasi-clique from protein networks. However, PPIs in biological organisms are complex, and do not depend only on a single feature, such as protein structural complementarity, gene proximity or co-evolution.

Moreover, some other protein interaction features, including sub-cellular localization, tissue specificity, cell-cycle stage and domain-domain combinations, are also critical factors to be considered. This study describes a scoring method based on integrating these heterogeneous but significant biological resources to prioritize human protein-protein interacting networks. The analytical results indicate that the proposed method can predict potential human PPIs with higher confidence than the other methods studied (Figure 2). The analytical results also reveal that some correlations exist between the true positive data set and the data set produced by the proposed method (Figure 3). Furthermore, the conservation score of a true positive interaction data set is higher that the score of the putative interaction data set (Table 2). Additionally, the proposed method allows researchers to identify quantitatively, rather than simply qualitatively, how (functional domain), when (cell cycle stage) and where (cellular compartment and tissue specificity) the two proteins interact, using a confidence score.

## Methods

Some studies have been published on the experimental derivation of PPIs and so does the *in silico *PPIs. Examples of topics examined include domain-domain co-occurrence [31,47,48], gene co-expression as shown by microarrays [49-52] and co-localization to the same sub-cellular compartment using Gene Ontology cellular component terms [35,38,53,54]. The combination of such evidence can support a broader range of PPIs than the predicted results from any single feature.

**Figure 5:**
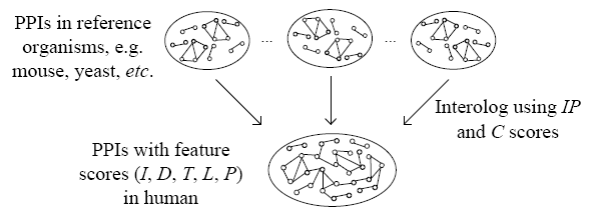
**Schematic illustration of scoring method for human PPIs determined from interologs**. The protein pair (a, b) is a known interaction in the reference organism, and the corresponding orthologous protein pair (A, B) can be inferred to interact in the target organism. The five-tuple score (*I*, *D*, *T*, *L*, *P*) is normalized to obtain a confidence score (*CS*).

Protein-protein interactions can be represented as a network graph whose vertices are proteins. These vertices are linked by edges if the corresponding proteins interact. In this study, the maximal quasi-clique determines a conservation score (*C*) from reference to target organism, and the interolog score (*I*) from the orthologous scores (*IP*) and (*C*). The other features of the protein interaction, such as spatial proximity (sub-cellular localization (*L*) and tissue-specificity (*T*)), temporal synchronicity (cell-cycle phase (*P*)) and domain-domain combinations (*D*) are also considered. Each score is normalized, and then these scores are summed into the final confidence score (*CS*). Figure 5 shows schematically the proposed scoring method.

### InParanoid score (*IP*)

The InParanoid [55] algorithm was designed to distinguish potential true orthologs from co-orthologs (paralogs) based on the best pairwise protein sequence similarity between organisms. The orthologous score, *IP *denotes the InParanoid score; the main orthologs always receive a score of 1.0, and the other paralogs receive scores from 0.0 to 1.0. Table 8 shows the predicted interologs and true positives mapped using only InParanoid data without considering other features. A lower *IP *score indicates more true positives in quantity. This finding indicates that the ortholog mappings across species are one-to-many and many-to-many. However, it also reveals that the true positive ratio does not signify an improvement in quality. The other features must be considered in order to filter out the predicted interactions that have low confidence scores.

**Table 8:** Number of interologs and true positives predicted by InParanoid score (*IP*) without other feature scores.

InParanoid score	Predicted interologs	True positives	Putatives	Precision	Recall
IP > 0.0	90,871	2,572	88,299	2.83%	-*
*IP *> 0.1	82,529	2,489	80,040	3.02%	96.77%
*IP *≥ 0.2	72,266	2,368	69,898	3.28%	92.07%
*IP *≥ 0.3	66,764	2,305	64,459	3.45%	89.62%
*IP *≥ 0.4	60,916	2,214	58,702	3.63%	86.08%
*IP *≥ 0.5	54,481	2,134	52,347	3.92%	82.97%
*IP *≥ 0.6	49,778	2,069	47,709	4.16%	80.44%
*IP *≥ 0.7	44,531	1,986	42,545	4.46%	77.22%
*IP *≥ 0.8	41,354	1,958	39,396	4.73%	76.13%
*IP *≥ 0.9	38,984	1,930	37,054	4.95%	75.04%
*IP *= 1.0	36,376	1,918	34,458	5.27%	74.57%

* : the predicted data set do not contain negative data to calculate false negative (FN) to obtain recall = (TP/(TP+FN))

### Quasi-clique and conservation score (*C*)

Let *G *= (*V*, *E*) denote a graph, where *V *is the set of vertices, and *E *is the set of edges in graph *G*. A graph is *γ*-dense, such that *γ *= 2 |*E*|/|*V*| (|*V*| - 1). For a subset *S *⊆ *V*, *G*^*S *^is the sub-graph induced by *S*. A quasi-clique, also called a *γ*-clique *S*, is a subset of *G*, such that the induced graph *G*^*S *^is connected and *γ*-clique. The original maximum problem *γ*-clique *S *is to find a 1-clique, complete sub-graph (*γ *= 1) with maximum vertices in graph *G*.

A quasi-clique in PPI networks is a group of proteins that tend to interact with each other, but a complete sub-graph (*γ *= 1) is not always biologically significant. Hence, *C *= *γ *|*E*| is defined as the protein complex conservation score. The value of |*E*| is the functional links of a protein complex.

**Figure 6:**
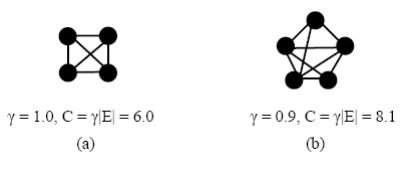
**Relationship among *γ*, |*E*| and *C***. Relationship among *γ*, |*E*| and *C*. (a) Three proteins interacting as a complex with three functional links; (b) five proteins interacting as a complex with nine functional links. Although the protein complex in (a) has a higher *γ *= 1.0 than the protein complex in (b), that in (b) is more biologically significant. Therefore,*C *= *γ *|*E*| is taken as the relative conservation score for a protein complex.

Some recent studies have concluded that motif modules and their constituents in a specific functional protein network are highly conserved across species [56,57]. Evolutionary rate analysis [58] has indicated that the connectivity of well-conserved proteins in the network is negatively correlated with their rate of evolution. More connected proteins in an interaction network evolve at a lower rate, because they are subject to a higher pressure to co-evolve with other interacting proteins. This study searches for a quasi-clique with maximal relative conservation score *C *in a protein complex. Figure 6 illustrates an example of such a quasi-clique.

### Interolog score (*I*)

The protein interaction bases utilized for mapping human protein interaction networks were obtained from six eukaryotes, namely *Rattus norvegicus, Mus musculus, Drosophila melanogaster, Caenorhabditis elegans, Arabidopsis thaliana *and *Saccharomyces cerevisiae*, as reference organisms. These data were obtained from AfCS-Nature [59], BIND, BioGRID, CYGD, CORE subset of DIP, IntAct, MINT and MPPI. Table 9 lists the numbers of distinct interactions in each data set.

**Table 9:** Number and sources of model organism interaction data sets.

		Organisms						
		
	Version	Human	Rat	Mouse	Fly	Worm	Thale cress	Baker's yeast
AfCS-Nature	2005/10/14	-	-	763	-	-	-	-
BIND	2005/07/10	1,755	317	1,077	15,693	3,417	-	11,502
BioGRID	2.0.20	15,578	-	-	18,919	4,921	-	48,011
CYGD	2006/05/18	-	-	-	-	-	-	11,778
DIP	2006/04/02	703	20	61	564	2,371	-	5,067
HPRD	2006/01/06	18,767	-	-	-	-	-	-
IntAct	2006/06/16	7,046	854	1,464	22,322	4,585	2,134	74,961
MINT	2005/05/20	3,236	138	767	18,573	3,970	-	11,223
MPPI	2005/04/25	247	83	185	-	-	-	-
Rual *et, al*	[39]	4,044	-	-	-	-	-	-
Stelzl *et, al*	[40]	2,889	-	-	-	-	-	-

Total (nr)	-	37,929	1,344	3,895	44,119	7,690	2,134	115,903

**Figure 8:**
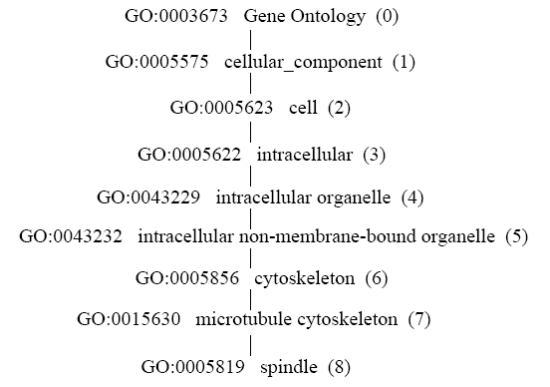
**Example of GO cellular component hierarchy from depth levels 0 to 8**. A protein pair (A, B) with GO cellular component annotations 'cell' and 'spindle' at depths 2 and 8, respectively. The common GO terms among their ancestor terms (including the original terms) are 'Gene Ontology', 'cellular component' and 'cell'. The deepest term is 'cell', at a depth of 2.

The interolog concept states that proteins that interact in a single organism co-evolve so that their respective orthologs maintain the ability to interact in another organism. For example, as shown in Figure 8, if two proteins (a, b) interact in the reference organism, then the corresponding pairs of orthologs and paralogs (*A*_1_, *B*_1_), (*A*_1_, *B*_2_), (*A*_1_, *B*_3_), (*A*_2_, *B*_1_), (*A*_2_, *B*_2_) and (*A*_2_, *B*_3_) can be inferred to interact in a target organism, and the interolog score (*I*) can be determined as follows.

Iij=wec∗min⁡(IPAi,IPBj)∗Cab
 MathType@MTEF@5@5@+=feaafiart1ev1aaatCvAUfKttLearuWrP9MDH5MBPbIqV92AaeXatLxBI9gBaebbnrfifHhDYfgasaacH8akY=wiFfYdH8Gipec8Eeeu0xXdbba9frFj0=OqFfea0dXdd9vqai=hGuQ8kuc9pgc9s8qqaq=dirpe0xb9q8qiLsFr0=vr0=vr0dc8meaabaqaciaacaGaaeqabaqabeGadaaakeaacqWGjbqsdaWgaaWcbaGaemyAaKMaemOAaOgabeaakiabg2da9iabdEha3naaBaaaleaacqWGLbqzcqWGJbWyaeqaaOGaey4fIOIagiyBa0MaeiyAaKMaeiOBa4MaeiikaGIaemysaKKaemiuaa1aaSbaaSqaaiabbgeabnaaBaaameaacqqGPbqAaeqaaaWcbeaakiabcYcaSiabdMeajjabdcfaqnaaBaaaleaacqqGcbGqdaWgaaadbaGaeeOAaOgabeaaaSqabaGccqGGPaqkcqGHxiIkcqWGdbWqdaWgaaWcbaGaeeyyaeMaeeOyaigabeaaaaa@4C97@

The weight of evolutionary conservation (*w*_*ec*_) is defined such that a higher *w*_*ec *_value indicates an organism that is genetically closer to humans. The following *w*_*ec *_values were considered: *w*_rat _= 1.0, *w*_mouse _= 1.0, *w*_fly _= 0.75, *w*_worm _= 0.75, *w*_thalecress _= 0.5 and *w*_yeast _= 0.25 for rat, mouse, fly, worm, thale cress and baker's yeast, respectively. Because rat and mouse are both mammals, and are thus genetically closest to human, they were assigned the highest value of 1.0. *Drosophila *and *C. elegans *are two animal models that are widely studied to understand human disease genes and development, and are ranked second closest to humans among the organisms studied. Finally, thale cress is sorted in higher order than yeast, since it is multi-cellular organism, while yeast is a single-cell species. If a pair of human protein interactions is derived from two or more reference model organisms, then only the highest interolog score is used to generate non-redundant (nr) human protein-protein interactions.

### Domain-domain combination score (*D*)

A probabilistic framework [31] has been presented to predict the interaction probability of proteins, and an interaction possibility ranking method has been developed for multiple protein pairs using the Potentially Interacting Domain Combination Pair (PIDC). This study utilized the concept of PIDC, collecting all domain combinations were accumulated from the known interactions in the experimental databases. A pair of interacting proteins A and B with multiple domains was obtained. For example, a domain set *D*_d _= {*d*_1_, *d*_2_, *d*_3_, ..., *d*_*m*_}, and its power set *PD*_d _= {{*d*_1_}, {*d*_2_}, {*d*_3_}, ..., {*d*_1_, *d*_2_, *d*_3_, ..., *d*_*m*_}}. The protein domain information was downloaded from the Pfam [60] domain annotation database. The domain-domain combination score, *D*, was calculated by summing the appearance probability as follows:

D=∑j=12m−1∑i=12m−1N′(pdi,pdj)N(pdi,pdj) if pdi∈PDd,pdj∈PDd
 MathType@MTEF@5@5@+=feaafiart1ev1aaatCvAUfKttLearuWrP9MDH5MBPbIqV92AaeXatLxBI9gBaebbnrfifHhDYfgasaacH8akY=wiFfYdH8Gipec8Eeeu0xXdbba9frFj0=OqFfea0dXdd9vqai=hGuQ8kuc9pgc9s8qqaq=dirpe0xb9q8qiLsFr0=vr0=vr0dc8meaabaqaciaacaGaaeqabaqabeGadaaakeaacqWGebarcqGH9aqpdaaeWbqaamaaqahabaWaaSaaaeaacuWGobGtgaqbaiabcIcaOiabdchaWjabdsgaKnaaBaaaleaacqqGPbqAaeqaaOGaeiilaWIaemiCaaNaemizaq2aaSbaaSqaaiabbQgaQbqabaGccqGGPaqkaeaacqWGobGtcqGGOaakcqWGWbaCcqWGKbazdaWgaaWcbaGaeeyAaKgabeaakiabcYcaSiabdchaWjabdsgaKnaaBaaaleaacqqGQbGAaeqaaOGaeiykaKcaaaWcbaGaemyAaKMaeyypa0JaeGymaedabaGaeGOmaiZaaWbaaWqabeaacqWGTbqBaaWccqGHsislcqaIXaqma0GaeyyeIuoaaSqaaiabdQgaQjabg2da9iabigdaXaqaaiabikdaYmaaCaaameqabaGaemyBa0gaaSGaeyOeI0IaeGymaedaniabggHiLdGccqqGGaaicqqGPbqAcqqGMbGzcqqGGaaicqWGWbaCcqWGKbazdaWgaaWcbaGaeeyAaKgabeaakiabgIGiolabdcfaqjabdseaenaaBaaaleaacqqGKbazaeqaaOGaeiilaWIaemiCaaNaemizaq2aaSbaaSqaaiabbQgaQbqabaGccqGHiiIZcqWGqbaucqWGebardaWgaaWcbaGaeeizaqgabeaaaaa@73A7@

where *pd*_i _and *pd*_j _are sets *i *and *j *in the power set *PD*_d_, respectively, and *N' *(*pd*_i_, *pd*_j_) and *N *(*pd*_i_, *pd*_j_) are the number of interacting protein pairs and the total number of protein pairs that contain (*pd*_i_, *pd*_j_) in known interactions, respectively.

### Tissue specificity score (*T*)

The tissue specificity is another spatial proximity value to be considered. Two proteins that are activated at the same sub-cellular localization, and co-expressed in the same tissue, are likely to interact with each other. This information can be used to discover tissue-specific PPIs associated with human diseases for biomedical research. Tissue-specific gene expression information was extracted from the GeneAtlas Affymetrix data set, which includes 44, 775 human probe sets (30, 694 proteins) from 79 normal human tissue samples [61].

Score *T *denotes the tissue specificity score, calculated by summing the number of common tissues if two proteins both have 2-fold up-regulated expressions (log_2 _expression ratio = 1) than the mean expression value of specific tissue.

T=∑i=1791 if log⁡2eAieA¯≥1 and log⁡2eBieB≥1
 MathType@MTEF@5@5@+=feaafiart1ev1aaatCvAUfKttLearuWrP9MDH5MBPbIqV92AaeXatLxBI9gBaebbnrfifHhDYfgasaacH8akY=wiFfYdH8Gipec8Eeeu0xXdbba9frFj0=OqFfea0dXdd9vqai=hGuQ8kuc9pgc9s8qqaq=dirpe0xb9q8qiLsFr0=vr0=vr0dc8meaabaqaciaacaGaaeqabaqabeGadaaakeaacqWGubavcqGH9aqpdaaeWbqaaiabigdaXaWcbaGaemyAaKMaeyypa0JaeGymaedabaGaeG4naCJaeGyoaKdaniabggHiLdGccqqGGaaicqqGPbqAcqqGMbGzcqqGGaaicyGGSbaBcqGGVbWBcqGGNbWzdaWgaaWcbaGaeGOmaidabeaakmaalaaabaGaemyzauMaeeyqae0aaSbaaSqaaiabdMgaPbqabaaakeaadaqdaaqaaiabdwgaLjabdgeabbaaaaGaeyyzImRaeGymaeJaeeiiaaIaeeyyaeMaeeOBa4MaeeizaqMaeeiiaaIagiiBaWMaei4Ba8Maei4zaC2aaSbaaSqaaiabikdaYaqabaGcdaWcaaqaaiabdwgaLjabbkeacnaaBaaaleaacqWGPbqAaeqaaaGcbaGaemyzauMaemOqaieaaiabgwMiZkabigdaXaaa@5DF2@

where *e*A_*i *_and *e*B_*i *_are the normalized expression values of proteins A and B, respectively, in tissue sample *i*, and eA¯=∑i=179eAi
 MathType@MTEF@5@5@+=feaafiart1ev1aaatCvAUfKttLearuWrP9MDH5MBPbIqV92AaeXatLxBI9gBaebbnrfifHhDYfgasaacH8akY=wiFfYdH8Gipec8Eeeu0xXdbba9frFj0=OqFfea0dXdd9vqai=hGuQ8kuc9pgc9s8qqaq=dirpe0xb9q8qiLsFr0=vr0=vr0dc8meaabaqaciaacaGaaeqabaqabeGadaaakeaadaqdaaqaaiabdwgaLjabdgeabbaacqGH9aqpdaaeWaqaaiabdwgaLjabbgeabnaaBaaaleaacqWGPbqAaeqaaaqaaiabdMgaPjabg2da9iabigdaXaqaaiabiEda3iabiMda5aqdcqGHris5aaaa@3B48@ and eB¯=∑i=179eBi
 MathType@MTEF@5@5@+=feaafiart1ev1aaatCvAUfKttLearuWrP9MDH5MBPbIqV92AaeXatLxBI9gBaebbnrfifHhDYfgasaacH8akY=wiFfYdH8Gipec8Eeeu0xXdbba9frFj0=OqFfea0dXdd9vqai=hGuQ8kuc9pgc9s8qqaq=dirpe0xb9q8qiLsFr0=vr0=vr0dc8meaabaqaciaacaGaaeqabaqabeGadaaakeaadaqdaaqaaiabdwgaLjabdkeacbaacqGH9aqpdaaeWaqaaiabdwgaLjabbkeacnaaBaaaleaacqWGPbqAaeqaaaqaaiabdMgaPjabg2da9iabigdaXaqaaiabiEda3iabiMda5aqdcqGHris5aaaa@3B4C@ are the mean expression values of proteins A and B, respectively, under 79 tissue samples.

### Sub-cellular localization score (*L*)

The physical PPI requires contact between two proteins at certain cellular locations. Hence, this study used the Gene Ontology (GO) [62] annotation in the deep 'Cellular Component' (CC) hierarchy, discarding irrelevant GO terms such as 'cellular component unknown' and 'obsolete cellular component'.

If two interacting proteins share a common ancestor of the GO term, then *L *is the sub-cellular localization score, which is the deepest level number of the common GO term among ancestor terms (including itself) in the GO hierarchy. For example, a protein pair (A, B) has the GO cellular component annotation 'GO:0005623 cell' and 'GO:0005819 spindle' at depths of 2 and 8, respectively. The sub-cellular localization score *L *= 2 since the deepest level of common GO term among ancestors is at a depth of 2 in the GO hierarchy. Figure 8 shows the detailed hierarchy.

### Cell-cycle stage score (*P*)

Human cell cycle cDNA microarray analysis [63] reveals cell cycle-regulated genes. Table 10 lists the numbers of non-redundant (nr) proteins mapped from the original 1, 134 expressed clones at different cell cycle phases. The cell-development stage score *P *is given by the number of cell cycle phases in the overlap between two interacting proteins.

**Table 10:** Number of human cell cycle-regulated proteins at different phases.

Cell cycle stage	Expressed clones	Proteins
G1/S	211	137
S	221	146
G2	239	160
G2/M	273	208
M/G1	190	137

Total (nr)	1,134	788

### Confidence score (*CS*)

The five-tuple score (*I*, *D*, *T*, *L*, *P*) is an overall confidence score determined from equation (8), where the DK¯
 MathType@MTEF@5@5@+=feaafiart1ev1aaatCvAUfKttLearuWrP9MDH5MBPbIqV92AaeXatLxBI9gBaebbnrfifHhDYfgasaacH8akY=wiFfYdH8Gipec8Eeeu0xXdbba9frFj0=OqFfea0dXdd9vqai=hGuQ8kuc9pgc9s8qqaq=dirpe0xb9q8qiLsFr0=vr0=vr0dc8meaabaqaciaacaGaaeqabaqabeGadaaakeaadaqdaaqaaiabdseaenaaBaaaleaacqWGlbWsaeqaaaaaaaa@2F19@, LK¯
 MathType@MTEF@5@5@+=feaafiart1ev1aaatCvAUfKttLearuWrP9MDH5MBPbIqV92AaeXatLxBI9gBaebbnrfifHhDYfgasaacH8akY=wiFfYdH8Gipec8Eeeu0xXdbba9frFj0=OqFfea0dXdd9vqai=hGuQ8kuc9pgc9s8qqaq=dirpe0xb9q8qiLsFr0=vr0=vr0dc8meaabaqaciaacaGaaeqabaqabeGadaaakeaadaqdaaqaaiabdYeamnaaBaaaleaacqWGlbWsaeqaaaaaaaa@2F29@, PK¯
 MathType@MTEF@5@5@+=feaafiart1ev1aaatCvAUfKttLearuWrP9MDH5MBPbIqV92AaeXatLxBI9gBaebbnrfifHhDYfgasaacH8akY=wiFfYdH8Gipec8Eeeu0xXdbba9frFj0=OqFfea0dXdd9vqai=hGuQ8kuc9pgc9s8qqaq=dirpe0xb9q8qiLsFr0=vr0=vr0dc8meaabaqaciaacaGaaeqabaqabeGadaaakeaadaqdaaqaaiabdcfaqnaaBaaaleaacqWGlbWsaeqaaaaaaaa@2F31@ and TK¯
 MathType@MTEF@5@5@+=feaafiart1ev1aaatCvAUfKttLearuWrP9MDH5MBPbIqV92AaeXatLxBI9gBaebbnrfifHhDYfgasaacH8akY=wiFfYdH8Gipec8Eeeu0xXdbba9frFj0=OqFfea0dXdd9vqai=hGuQ8kuc9pgc9s8qqaq=dirpe0xb9q8qiLsFr0=vr0=vr0dc8meaabaqaciaacaGaaeqabaqabeGadaaakeaadaqdaaqaaiabdsfaunaaBaaaleaacqWGlbWsaeqaaaaaaaa@2F39@ are the mean values of each feature score from known human interaction data sets (KNOWN2). IR¯
 MathType@MTEF@5@5@+=feaafiart1ev1aaatCvAUfKttLearuWrP9MDH5MBPbIqV92AaeXatLxBI9gBaebbnrfifHhDYfgasaacH8akY=wiFfYdH8Gipec8Eeeu0xXdbba9frFj0=OqFfea0dXdd9vqai=hGuQ8kuc9pgc9s8qqaq=dirpe0xb9q8qiLsFr0=vr0=vr0dc8meaabaqaciaacaGaaeqabaqabeGadaaakeaadaqdaaqaaiabdMeajnaaBaaaleaacqWGsbGuaeqaaaaaaaa@2F31@ is the mean interolog score in one reference organism.

CS=wI∗IIR¯+wD∗DDK¯+wT∗TTK¯+wL∗LLK¯+wP∗PPK¯
 MathType@MTEF@5@5@+=feaafiart1ev1aaatCvAUfKttLearuWrP9MDH5MBPbIqV92AaeXatLxBI9gBaebbnrfifHhDYfgasaacH8akY=wiFfYdH8Gipec8Eeeu0xXdbba9frFj0=OqFfea0dXdd9vqai=hGuQ8kuc9pgc9s8qqaq=dirpe0xb9q8qiLsFr0=vr0=vr0dc8meaabaqaciaacaGaaeqabaqabeGadaaakeaacqWGdbWqcqWGtbWucqGH9aqpcqWG3bWDdaWgaaWcbaGaemysaKeabeaakiabgEHiQmaalaaabaGaemysaKeabaWaa0aaaeaacqWGjbqsdaWgaaWcbaGaemOuaifabeaaaaaaaOGaey4kaSIaem4DaC3aaSbaaSqaaiabdseaebqabaGccqGHxiIkdaWcaaqaaiabdseaebqaamaanaaabaGaemiraq0aaSbaaSqaaiabdUealbqabaaaaaaakiabgUcaRiabdEha3naaBaaaleaacqWGubavaeqaaOGaey4fIOYaaSaaaeaacqWGubavaeaadaqdaaqaaiabdsfaunaaBaaaleaacqWGlbWsaeqaaaaaaaGccqGHRaWkcqWG3bWDdaWgaaWcbaGaemitaWeabeaakiabgEHiQmaalaaabaGaemitaWeabaWaa0aaaeaacqWGmbatdaWgaaWcbaGaem4saSeabeaaaaaaaOGaey4kaSIaem4DaC3aaSbaaSqaaiabdcfaqbqabaGccqGHxiIkdaWcaaqaaiabdcfaqbqaamaanaaabaGaemiuaa1aaSbaaSqaaiabdUealbqabaaaaaaaaaa@58CB@

In this scoring scheme, all data sources are weighted equally: *w*_*I *_= 1, *w*_*D *_= 1, *w*_*T *_= 1, *w*_*L *_= 1 and *w*_*P *_= 1. Moreover, the confidence score *CS *= 4, as derived by recall ratio ≥ 50% (Table 6).

## Abbreviations

KNONW – Human known interaction data set obtained from well-known databases.

KNONW2 – KNOWN2 is derived from KNOWN with addition of two experimental data [39,40].

TP0 – The overlapping of KNOWN2 and our predicted data set when confidence score *CS *> 0.

PU0 – PU0 is the all-predicted data set absent from TP0 when confidence score *CS *> 0.

TP4 – The overlap of KNOWN2 and the predicted data set when confidence score *CS *≥ 4.

PU4 – The all predicted data set absent from TP4 when confidence score *CS *≥ 4.

BTP – The overlap of KNOWN2 and BLAST predicted data sets.

BPU – The all BLAST predicted data set absent from BTP.

RANDOMS – Random interaction data sets with the same number of TP4 interactions.

MF – Molecular function in Gene Ontology categories.

BP – Biological process in Gene Ontology categories.

*C *– Conservation score.

*IP *– InParanoid score.

*I *– Interolog score.

*D *– Domain-domain combination score.

*T *– Tissue specific score.

*L *– Sub-cellular localization score.

*P *– Cell-cycle stage score.

*CS *– Confidence score.

## Authors' contributions

All authors participated in the development of the methodology in the manuscript. All authors read and approved the final version of the manuscript.

**Figure 7:**
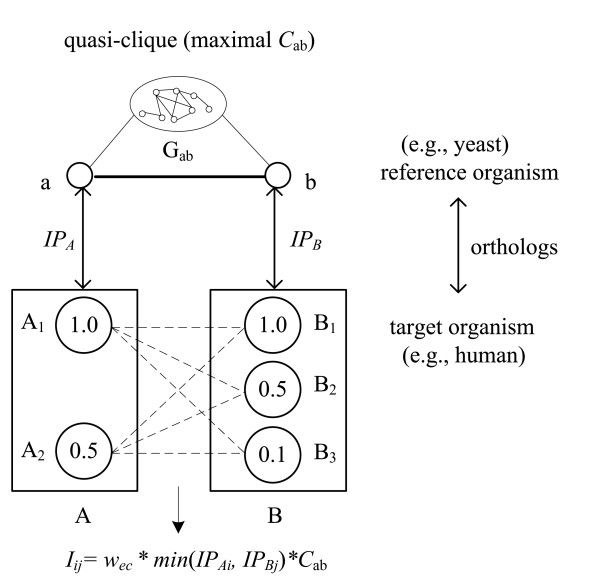
**Protein-protein interolog score**. Protein-protein interolog score, where A-a and B-b are orthologs between the two organisms. The orthologous protein pair (*A*_*i*_, *B*_*j*_) can be inferred to interact in a target organism if the protein pair (a, b) interacts in a reference organism. *G*_ab _is the sub-graph of proteins that interact with both a and b; *C*_ab _is the quasi-clique with maximal conservation score in *G*_ab_, and *IP*_Ai _and *IP*_Bj _are the InParanoid scores of paralogs *i *and *j *of orthologs A and B, respectively, in the target organism.

## Supplementary Material

Additional file 1 **Data set of all predicted protein-protein interaction**. A plain text with tab-delimited format. Column 1 through 12 are two protein UniProt IDs, two protein, InParanoid (*IP*) scores, normalized *C*, *I*, *D*, *T*, *L*, *P*, *CS *and *p*-value, respectively. Click here for file

Additional file 2 **Distributions of the various components of the confidence metrics and ANOVA tests between different interaction data sets**. This file contains distributions of the various feature scores (*D, T, L, P*) and ANOVA tests between different interaction data sets, i.e. KNOWN2, TP4, TP0, BTP, PU4, PU0, BPU and RANDOMS. Click here for file

Additional file 3 **Top 20 total predicted putative interacting protein pairs not present in existing experimental data sets (KNOWN2)**. The top 20 predicted putative interacting protein pairs are listed with their OMIM ID, GO 'molecular function' and 'biological process' annotations and UniProt functional keywords. Click here for file
