# Supplementary material for: Reconstruction of human protein interolog network using evolutionary conserved network
Source: BMC Bioinformatics. 2007 May 10;8:152. doi: 10.1186/1471-2105-8-152 (PMC1885812; doi:10.1186/1471-2105-8-152)
Supplement: Additional file 2 — Distributions of the various components of the confidence metrics and ANOVA tests between different interaction data sets. This file contains distributions of the various feature scores (D, T, L, P) and ANOVA tests between different interaction data sets, i.e. KNOWN2, TP4, TP0, BTP, PU4, PU0, BPU and RANDOMS. [file 1471-2105-8-152-S2.pdf]

Additional File 2

Additional Table A1 — Distributions of D score between the different interaction data sets

	Interaction data sets							
D Score	KNOWN2	TP4	TP0	BTP	PU4	PU0	BPU	RANDOMS
Min.	0.00000	0.00000	0.00000	0.00000	0.00000	0.00000	0.00000	0.00000
Mean	0.00035	0.00080	0.00050	0.00040	0.00030	0.00011	0.00009	0.00003
Max.	0.02165	0.01589	0.01589	0.01589	0.01783	0.01783	0.01783	0.01783
SD.	0.00117	0.00182	0.00141	0.00108	0.00109	0.00053	0.00033	0.00019
Std Err	0.00001	0.00005	0.00003	0.00002	0.00001	0.00000	0.00000	0.00000

Additional Table A2 — ANOVA tests of D score between the different interaction data sets

Comparison	Diff of Means	t	Unadjusted P	Critical Level	Significant?
KNOWN2 vs. TP4	0.000452	27.06	4.43E-161	0.004	Yes
KNOWN2 vs. TP0	0.000148	11.609	3.78E-31	0.009	Yes
KNOWN2 vs. BTP	0.0000499	4.877	0.00000108	0.05	Yes
KNOWN2 vs. PU4	0.0000483	8.776	1.70E-18	0.013	Yes
KNOWN2 vs. PU0	0.00025	63.99000	0.00000	0.00200	Yes
KNOWN2 vs. BPU	0.00026	67.46400	0.00000	0.00200	Yes
KNOWN2 vs. RANDOMS	0.00032	83.31700	0.00000	0.00200	Yes
TP4 vs. TP0	0.000304	14.818	1.17E-49	0.007	Yes
TP4 vs. BTP	0.000402	21.085	1.26E-98	0.005	Yes
TP4 vs. PU4	0.0005	29.446	2.50E-190	0.004	Yes
TP4 vs. PU0	0.000697	42.223	0	0.002	Yes
TP4 vs. BPU	0.000714	43.227	0	0.003	Yes
TP4 vs. RANDOMS	0.00077	46.64300	0.00000	0.00200	Yes
TP0 vs. BTP	0.0000979	6.235	4.51E-10	0.017	Yes
TP0 vs. PU4	0.000196	14.961	1.37E-50	0.006	Yes
TP0 vs. PU0	0.000393	31.464	5.69E-217	0.003	Yes
TP0 vs. BPU	0.000411	32.789	2.03E-235	0.003	Yes
TP0 vs. RANDOMS	0.000466	37.302	6.80E-304	0.003	Yes
BTP vs. PU4	0.0000983	9.179	4.38E-20	0.01	Yes
BTP vs. PU0	0.000295	29.696	1.59E-193	0.004	Yes
BTP vs. BPU	0.000313	31.349	2.11E-215	0.003	Yes
BTP vs. RANDOMS	0.000368	37.035	1.33E-299	0.003	Yes
PU4 vs. PU0	0.000197	39.907	0	0.002	Yes
PU4 vs. BPU	0.00021	42.99200	0.00000	0.00200	Yes
PU4 vs. RANDOMS	0.00	54.78	0.00	0.00	Yes
PU0 vs. BPU	0.0000171	5.624	1.86E-08	0.025	Yes
PU0 vs. RANDOMS	0.0000728	24.647	5.24E-134	0.005	Yes
BPU vs. RANDOMS	0.0000557	18.39	1.72E-75	0.006	Yes

Additional Table A3 — Distributions of T score between the different interaction data sets

	Interaction data sets							
T score	KNOWN2	TP4	TP0	BTP	PU4	PU0	BPU	RANDOM
0	18,107 (47.74%)	151 (10.29%)	672 (26.13%)	1,239 (30.00%)	6246 (34.33%)	57,614 (65.25%)	42,203 (52.51%)	68,659 (75.56%)
1	5,344 (14.09%)	72 (4.91%)	320 (12.44%)	557 (13.49%)	1199 (6.59%)	8,730 (9.89%)	9,159 (11.40%)	8,839 (9.73%)
2	3,242 (8.55%)	60 (4.09%)	186 (7.23%)	343 (8.31%)	1115 (6.13%)	5,488 (6.22%)	6,273 (7.81%)	4,706 (5.18%)
3	2,269 (5.98%)	74 (5.04%)	166 (6.45%)	267 (6.46%)	1112 (6.11%)	3,987 (4.52%)	4,621 (5.75%)	2,838 (3.12%)
4	1,743 (4.60%)	68 (4.64%)	116 (4.51%)	170 (4.12%)	1054 (5.79%)	2,829 (3.20%)	3,399 (4.23%)	1,777 (1.96%)
5	1,339 (3.53%)	100 (6.82%)	124 (4.82%)	183 (4.43%)	1137 (6.25%)	2,203 (2.49%)	2,926 (3.64%)	1,236 (1.36%)
6	1,047 (2.76%)	83 (5.66%)	111 (4.32%)	155 (3.75%)	1009 (5.55%)	1,651 (1.87%)	2,384 (2.97%)	812 (0.89%)
7	853 (2.25%)	79 (5.39%)	87 (3.38%)	139 (3.37%)	896 (4.93%)	1,185 (1.34%)	1,843 (2.29%)	615 (0.68%)
8	705 (1.86%)	93 (6.34%)	103 (4.00%)	140 (3.39%)	851 (4.68%)	1,007 (1.14%)	1,458 (1.81%)	384 (0.42%)
9	592 (1.56%)	84 (5.73%)	84 (3.27%)	113 (2.74%)	766 (4.21%)	798 (0.90%)	1,276 (1.59%)	314 (0.35%)
10	480 (1.27%)	81 (5.52%)	81 (3.15%)	107 (2.59%)	546 (3.00%)	546 (0.62%)	937 (1.17%)	213 (0.23%)
11	406 (1.07%)	67 (4.57%)	67 (2.60%)	92 (2.23%)	565 (3.11%)	565 (0.64%)	862 (1.07%)	151 (0.17%)
12	385 (1.02%)	59 (4.02%)	59 (2.29%)	87 (2.11%)	419 (2.30%)	419 (0.47%)	734 (0.91%)	99 (0.11%)
13	251 (0.66%)	52 (3.54%)	52 (2.02%)	73 (1.77%)	312 (1.72%)	312 (0.35%)	530 (0.66%)	65 (0.07%)
14	244 (0.64%)	43 (2.93%)	43 (1.67%)	64 (1.55%)	321 (1.76%)	321 (0.36%)	496 (0.62%)	61 (0.07%)
15	187 (0.49%)	46 (3.14%)	46 (1.79%)	64 (1.55%)	216 (1.19%)	216 (0.24%)	391 (0.49%)	25 (0.03%)
16	161 (0.42%)	35 (2.39%)	35 (1.36%)	56 (1.36%)	153 (0.84%)	153 (0.17%)	270 (0.34%)	25 (0.03%)
17	125 (0.33%)	45 (3.07%)	45 (1.75%)	59 (1.43%)	95 (0.52%)	95 (0.11%)	234 (0.29%)	20 (0.02%)
18	102 (0.27%)	35 (2.39%)	35 (1.36%)	47 (1.14%)	85 (0.47%)	85 (0.10%)	172 (0.21%)	16 (0.02%)
19	94 (0.25%)	34 (2.32%)	34 (1.32%)	42 (1.02%)	42 (0.23%)	42 (0.05%)	106 (0.13%)	7 (0.01%)
20	63 (0.17%)	13 (0.89%)	13 (0.51%)	26 (0.63%)	31 (0.17%)	31 (0.04%)	57 (0.07%)	6 (0.01%)
21	34 (0.09%)	23 (1.57%)	23 (0.89%)	26 (0.63%)	10 (0.05%)	10 (0.01%)	21 (0.03%)	3 (0.00%)
22	36 (0.09 %)	15 (1.02%)	15 (0.58%)	18 (0.44%)	3 (0.02%)	3 (0.00%)	12 (0.01%)	0 (0.00%)
23	40 (0.11 %)	14 (0.95%)	14 (0.54%)	16 (0.39%)	4 (0.02%)	4 (0.00%)	3 (0.00%)	0 (0.00%)
24	18 (0.05%)	11 (0.75%)	11 (0.43%)	12 (0.29%)	0 (0.00%)	0 (0.00%)	1 (0.00%)	0 (0.00%)
25	19 (0.05%)	12 (0.82%)	12 (0.47%)	12 (0.29%)	0 (0.00%)	0 (0.00%)	1 (0.00%)	0 (0.00%)
26	9 (0.02%)	3 (0.20%)	3 (0.12%)	5 (0.12%)	2 (0.01%)	2 (0.00%)	1 (0.00%)	0 (0.00%)
27	7 (0.02%)	2 (0.14%)	2 (0.08%)	3 (0.07%)	1 (0.01%)	1 (0.00%)	0 (0.00%)	0 (0.00%)
28	8 (0.02%)	4 (0.27%)	4 (0.16%)	7 (0.17%)	1 (0.01%)	1 (0.00%)	0 (0.00%)	0 (0.00%)
29	7 (0.02%)	0 (0.00%)	0 (0.00%)	2 (0.05%)	1 (0.01%)	1 (0.00%)	1 (0.00%)	0 (0.00%)
30	2 (0.01%)	2 (0.14%)	2 (0.08%)	1 (0.02%)	0 (0.00%)	0 (0.00%)	0 (0.00%)	0 (0.00%)
31	4 (0.01%)	5 (0.34%)	5 (0.19%)	3 (0.07%)	0 (0.00%)	0 (0.00%)	0 (0.00%)	0 (0.00%)
32	3 (0.01%)	2 (0.14%)	2 (0.08%)	2 (0.05%)	0 (0.00%)	0 (0.00%)	0 (0.00%)	0 (0.00%)
33	1 (0.00%)	0 (0.00%)	0 (0.00%)	0 (0.00%)	0 (0.00%)	0 (0.00%)	0 (0.00%)	0 (0.00%)
34	0 (0.00%)	0 (0.00%)	0 (0.00%)	0 (0.00%)	0 (0.00%)	0 (0.00%)	0 (0.00%)	0 (0.00%)
35	0 (0.00%)	0 (0.00%)	0 (0.00%)	0 (0.00%)	0 (0.00%)	0 (0.00%)	0 (0.00%)	0 (0.00%)
36	2 (0.01%)	0 (0.00%)	0 (0.00%)	0 (0.00%)	0 (0.00%)	0 (0.00%)	0 (0.00%)	0 (0.00%)
Total	37,929 (100.00%)	1,467 (100%)	2,572 (100%)	4,130 (100%)	18,192 (100%)	88,299 (100%)	80,371 (100%)	90,871 (100%)
Min.	0.000	0.000	0.000	0.000	0.000	0.000	0.000	0.000
Mean	2.453	8.973	5.612	4.897	4.104	1.398	2.201	0.699
Max.	36.000	32.000	32.000	32.000	29.000	29.000	29.000	21.000
Std Err	0.021	0.174	0.126	0.094	0.033	0.010	0.013	0.006

Additional Table A4 — ANOVA tests of T score between the different interaction data sets

Comparison	Diff of Means	t	Unadjusted P	Critical Level	Significant?
KNOWN2 vs. TP4	6.52	75.62	0	0.00	Yes
KNOWN2 vs. TP0	3.159	48.01	0	0.01	Yes
KNOWN2 vs. BTP	2.444	46.17	0	0.00	Yes
KNOWN2 vs. PU4	1.651	58.03	0	0.00	Yes
KNOWN2 vs. PU0	1.055	53.21	0	0.00	Yes
KNOWN2 vs. BPU	0.252	12.51	6.83E-36	0.03	Yes
KNOWN2 vs. RANDOMS	1.754	88.84	0	0.00	Yes
TP4 vs. TP0	3.361	31.74	1.07E-220	0.01	Yes
TP4 vs. BTP	4.077	41.42	0	0.01	Yes
TP4 vs. PU4	4.869	55.52	0	0.01	Yes
TP4 vs. PU0	7.575	88.80	0	0.01	Yes
TP4 vs. BPU	6.772	79.32	0.00E+00	0.00	Yes
TP4 vs. RANDOMS	8.274	97.02	0	0.00	Yes
TP0 vs. BTP	0.716	8.82	1.12E-18	0.05	Yes
TP0 vs. PU4	1.509	22.27	9.16E-110	0.01	Yes
TP0 vs. PU0	4.214	65.23	0	0.01	Yes
TP0 vs. BPU	3.411	52.72	0.00E+00	0.01	Yes
TP0 vs. RANDOMS	4.913	76.08	0	0.00	Yes
BTP vs. PU4	0.793	14.33	1.41E-46	0.02	Yes
BTP vs. PU0	3.499	68.04	0	0.00	Yes
BTP vs. BPU	2.695	52.30	0	0.00	Yes
BTP vs. RANDOMS	4.198	81.69	0	0.00	Yes
PU4 vs. PU0	2.706	105.95	0	0.00	Yes
PU4 vs. BPU	1.902	73.83	0	0.00	Yes
PU4 vs. RANDOMS	3.405	133.66	0	0.00	Yes
PU0 vs. BPU	0.803	51.03	0.00E+00	0.00	Yes
PU0 vs. RANDOMS	0.699	45.80	0.00E+00	0.00	Yes
BPU vs. RANDOMS	1.502	96.07	0	0.00	Yes

Additional Table A5 — Distributions of L score between the different interaction data sets

	Interaction data sets							
L score	KNOWN2	TP4	TP0	BTP	PU4	PU0	BPU	RANDOMS
1	11,818 (31.16%)	101 (6.88 %)	341 (13.26%)	668 (16.17%)	5,075 (27.90%)	44,125 (49.97%)	32,370 (40.28%)	55,496 (61.07%
2	4,084 (10.77%)	37 (2.52 %)	107 (4.16%)	209 (5.06%)	339 (1.86%)	4,538 (5.14%)	3,904 (4.86%)	8,975 (9.88%
3	2,778 (7.32%)	15 (1.02 %)	64 (2.49%)	164 (3.97%)	890 (4.89%)	5,542 (6.28%)	6,890 (8.57%)	6,063 (6.67%
4	7,320 (19.30%)	310 (21.13 %)	662 (25.74%)	1,077 (26.08%)	3,006 (16.52%)	9,983 (11.31%)	10,229 (12.73%)	5,434 (5.98%
5	1,563 (4.12%)	53 (3.61 %)	106 (4.12%)	157 (3.80%)	1,629 (8.95%)	5,528 (6.26%)	5,323 (6.62%)	2,620 (2.88%
6	59 (0.16%)	10 (0.68 %)	18 (0.70%)	18 (0.44%)	24 (0.13%)	73 (0.08%)	46 (0.06%)	7 (0.01%
7	1,312 (3.46%)	19 (1.30 %)	43 (1.67%)	84 (2.03%)	1,598 (8.78%)	5,658 (6.41%)	7,622 (9.48%)	6,296 (6.93%
8	7,456 (19.66%)	485 (33.06 %)	730 (28.38%)	1,064 (25.76%)	3,846 (21.14%)	10,418 (11.80%)	11,112 (13.83%)	5,857 (6.45%
9	436 (1.15%)	64 (4.36 %)	82 (3.19%)	136 (3.29%)	521 (2.86%)	934 (1.06%)	847 (1.05%)	61 (0.07%
10	361 (0.95%)	80 (5.45 %)	102 (3.97%)	138 (3.34%)	532 (2.92%)	651 (0.74%)	1,136 (1.41%)	38 (0.04%
11	207 (0.55%)	104 (7.09 %)	114 (4.43%)	141 (3.41%)	501 (2.75%)	565 (0.64%)	558 (0.69%)	16 (0.02%
12	339 (0.89%)	107 (7.29 %)	114 (4.43%)	135 (3.27%)	188 (1.03%)	225 (0.25%)	232 (0.29%)	6 (0.01%
13	120 (0.32%)	54 (3.68 %)	60 (2.33%)	93 (2.25%)	22 (0.12%)	35 (0.04%)	40 (0.05%)	0 (0.00%
14	17 (0.04%)	7 (0.48 %)	8 (0.31%)	16 (0.39%)	1 (0.01%)	2 (0.00%)	11 (0.01%)	0 (0.00%
15	46 (0.12%)	14 (0.95 %)	14 (0.54%)	26 (0.63%)	18 (0.10%)	20 (0.02%)	50 (0.06%)	2 (0.00%
16	7 (0.02%)	4 (0.27 %)	4 (0.16%)	3 (0.07%)	2 (0.01%)	2 (0.00%)	1 (0.00%)	0 (0.00%
17	5 (0.01%)	2 (0.14 %)	2 (0.08%)	0 (0.00%)	0 (0.00%)	0 (0.00%)	0 (0.00%)	0 (0.00%
18	1 (0.00%)	0 (0.00%)	0 (0.00%)	0 (0.00%)	0 (0.00%)	0 (0.00%)	0 (0.00%)	0 (0.00%
19	0 (0.00%)	1 (0.07%)	1 (0.04%)	1 (0.02%)	0 (0.00%)	0 (0.00%)	0 (0.00%)	0 (0.00%
Total	37,929 (100.00 %)	1,467 (100.00 %)	2,572 (100.00 %)	4,130 (100.00 %)	18,192 (100.00%)	88,299 (100.00 %)	80,371 (100.00 %)	90,871 (100.00 %
Min.	1.000	1.000	1.000	1.000	1.000	1.000	1.000	1.000
Mean	3.986	7.022	6.133	5.701	4.531	3.233	3.736	2.406
Max.	18.000	19.000	19.000	19.000	15.000	16.000	16.000	15.000
Std Err	0.015	0.090	0.069	0.054	0.023	0.009	0.010	0.008

Additional Table A6 — ANOVA tests of L score between the different interaction data sets

Comparison	Diff of Means	t	Unadjusted P	Critical Level	Significant?
KNOWN2 vs. TP4	3.036	41.38	0	0.00	Yes
KNOWN2 vs. TP0	2.147	38.34	7.950E-321	0.00	Yes
KNOWN2 vs. BTP	1.715	38.08	1.343E-316	0.01	Yes
KNOWN2 vs. PU4	0.545	22.52	3.22E-112	0.01	Yes
KNOWN2 vs. PU0	0.753	44.65	0	0.00	Yes
KNOWN2 vs. BPU	0.25	14.61	2.45E-48	0.02	Yes
KNOWN2 vs. RANDOMS	1.58	94.04	0	0.00	Yes
TP4 vs. TP0	0.889	9.87	5.65E-23	0.03	Yes
TP4 vs. BTP	1.321	15.78	4.60E-56	0.01	Yes
TP4 vs. PU4	2.491	33.38	7.84E-244	0.01	Yes
TP4 vs. PU0	3.789	52.21	0	0.00	Yes
TP4 vs. BPU	3.286	45.24	0.00E+00	0.00	Yes
TP4 vs. RANDOMS	4.616	63.61	0	0.00	Yes
TP0 vs. BTP	0.432	6.26	3.95E-10	0.05	Yes
TP0 vs. PU4	1.602	27.78	1.09E-169	0.01	Yes
TP0 vs. PU0	2.9	52.76	0.00E+00	0.00	Yes
TP0 vs. BPU	2.397	43.54	0	0.00	Yes
TP0 vs. RANDOMS	3.726	67.82	0.00E+00	0.00	Yes
BTP vs. PU4	1.17	24.85	3.26E-136	0.01	Yes
BTP vs. PU0	2.47	56.41	0.00	0.00	Yes
BTP vs. BPU	1.97	44.81	0.00	0.00	Yes
BTP vs. RANDOMS	3.295	75.35	0	0.00	Yes
PU4 vs. PU0	1.30	59.75	0.00	0.00	Yes
PU4 vs. BPU	0.795	36.27	1.71E-287	0.01	Yes
PU4 vs. RANDOMS	2.125	98.04	0	0.00	Yes
PU0 vs. BPU	0.503	37.56	4.28E-308	0.01	Yes
PU0 vs. RANDOMS	0.826	63.64	0.00E+00	0.00	Yes
BPU vs. RANDOMS	1.33	99.92	0.00E+00	0.00	Yes

Additional Table A7 — Distributions of P score between the different interaction data sets

	Interaction data sets							
P score	KNOWN2	TP4	TP0	BTP	PU4	PU0	BPU	RANDOMS
0	37,851 (99.79%)	1424 (97.06884799%)	2,529 (98.33%)	4,081 (98.81%)	18169 (99.8735708%)	88,276 (99.97%)	80,345 (99.97%)	90,867 (100.00%)
1	75 (0.20%)	41 (2.794819359%)	41 (1.59%)	47 (1.14%)	23 (0.1264292%)	23 (0.03%)	26 (0.03%)	4 (0.00%)
2	3 (0.01%)	2 (0.136332652%)	2 (0.08%)	2 (0.05%)	0 (0.00%)	0 (0.00%)	0 (0.00%)	0 (0.00%)
Total	37,929 (100%)	1,467 (100%)	2,572 (100%)	4,130 (100%)	18,192 (100%)	88,299 (100%)	80,371 (100%)	90,871 (100%)
Min.	0.000	0.000	0.000	0.000	0.000	0.000	0.000	0.000
Mean	0.002	0.031	0.017	0.012	0.001	0.000	0.000	0.000
Max.	2.000	2.000	2.000	2.000	1.000	1.000	1.000	1.000
Std Err	0.000	0.005	0.003	0.002	0.000	0.000	0.000	0.000

Additional Table A8 — ANOVA tests of P score between the different interaction data sets

Comparison	Diff of Means	t	Unadjusted P	Critical Level	Significant?
KNOWN2 vs. TP4	0.0287	34.715	1.44E-263	0.002	Yes
KNOWN2 vs. TP0	0.0154	24.301	2.49E-130	0.003	Yes
KNOWN2 vs. BTP	0.0102	20.092	9.84E-90	0.004	Yes
KNOWN2 vs. PU4	0.000958	3.506	0.000455	0.01	Yes
KNOWN2 vs. PU0	0.00188	9.846	7.22E-23	0.005	Yes
KNOWN2 vs. BPU	0.00181	9.377	6.85E-21	0.006	Yes
KNOWN2 vs. RANDOMS	0.00209	11.029	2.80E-28	0.005	Yes
TP4 vs. TP0	0.0134	13.163	1.47E-39	0.004	Yes
TP4 vs. BTP	0.0185	19.61	1.42E-85	0.004	Yes
TP4 vs. PU4	0.0297	35.262	7.30E-272	0.002	Yes
TP4 vs. PU0	0.03060	37.37500	0.00000	0.00200	Yes
TP4 vs. BPU	0.03060	37.26900	0.00000	0.00200	Yes
TP4 vs. RANDOMS	0.03080	37.64800	0.00000	0.00200	Yes
TP0 vs. BTP	0.00515	6.606	3.96E-11	0.006	Yes
TP0 vs. PU4	0.0163	25.078	1.18E-138	0.003	Yes
TP0 vs. PU0	0.0172	27.775	1.36E-169	0.002	Yes
TP0 vs. BPU	0.0172	27.635	6.55E-168	0.002	Yes
TP0 vs. RANDOMS	0.0175	28.136	5.83E-174	0.002	Yes
BTP vs. PU4	0.0112	21.026	4.45E-98	0.003	Yes
BTP vs. PU0	0.0121	24.476	3.49E-132	0.003	Yes
BTP vs. BPU	0.012	24.295	2.89E-130	0.003	Yes
BTP vs. RANDOMS	0.0123	24.93	4.71E-137	0.003	Yes
PU4 vs. PU0	0.000917	3.739	1.85E-04	0.009	Yes
PU4 vs. BPU	0.000854	3.451	0.000558	0.013	Yes
PU4 vs. RANDOMS	0.00113	4.633	3.60E-06	0.007	Yes
PU0 vs. BPU	0.000063	0.417	0.677	0.05	No
PU0 vs. RANDOMS	0.000216	1.477	0.14	0.025	No
BPU vs. RANDOMS	0.000279	1.861	0.0628	0.017	No
