# Supplementary material for: Reconstruction of human protein interolog network using evolutionary conserved network
Source: BMC Bioinformatics. 2007 May 10;8:152. doi: 10.1186/1471-2105-8-152 (PMC1885812; doi:10.1186/1471-2105-8-152)
Supplement: Additional file 3 — Top 20 total predicted putative interacting protein pairs not present in existing experimental data sets (KNOWN2). The top 20 predicted putative interacting protein pairs are listed with their OMIM ID, GO 'molecular function' and 'biological process' annotations and UniProt functional keywords. [file 1471-2105-8-152-S3.pdf]

Additional File 3 Top 20 predicted interacting protein pairs (from PU4 and PU0) not present in existing experimental data sets (KNOWN2).

Top 1	Protein A	Protein B
	STK6_HUMAN	PLK1_HUMAN
Molecular function GO term	GO:0004672;protein kinase activity;<br>GO:0005515;protein binding	GO:0004674;protein serine/threonine kinase activity;<br>GO:0005515;protein binding
Biological process GO term	GO:0006468;protein amino acid phosphorylation;<br>GO:0007049;cell cycle;<br>GO:0007051;spindle organization and biogenesis;<br>GO:0007067;mitosis;<br>GO:0031647;regulation of protein stability;<br>GO:0048015;phosphoinositide-mediated signaling	GO:0000074;regulation of progression through cell cycle;<br>GO:0007067;mitosis;<br>GO:0008283;cell proliferation
UniProt functional keyword matching	3D-structure<br>ATP-binding<br>Kinase<br>Nucleotide-binding<br>Phosphorylation<br>Serine/threonine-protein kinase<br>Transferase	
Cell-cycle stage	G2/M	
OMIM ID	602687	602098

Top 2	Protein A	Protein B
	ORC1_HUMAN	MCM4_HUMAN
Molecular function GO term	GO:0003677;DNA binding;<br>GO:0005515;protein binding	GO:0005515;protein binding;<br>GO:0005524;ATP binding
Biological process GO term	GO:0006270;DNA replication initiation	GO:0006260;DNA replication
UniProt functional keyword matching	ATP-binding;<br>DNA replication;<br>DNA-binding;<br>Nuclear protein;<br>Nucleotide-binding;<br>Polymorphism	
Cell-cycle stage	G1/S	
OMIM ID	601902	602638

Top 3	Protein A	Protein B
	MCM6_HUMAN	MCM5_HUMAN
Molecular function GO term	GO:0005524;ATP binding;<br>GO:0042802;identical protein binding	GO:0005515;protein binding
Biological process GO term	O:0006260;DNA replication	GO:0000074;regulation of progression through cell cycle;<br>GO:0006260;DNA replication
UniProt functional keyword matching	ATP-binding;<br>DNA replication;<br>DNA-binding;<br>Nuclear protein;<br>Nucleotide-binding;<br>Polymorphism;<br>Transcription;<br>Transcription regulation	
Cell-cycle stage	G1/S	
OMIM ID	601806	602696

Top 4	Protein A	Protein B
	MCM4_HUMAN	MCM5_HUMAN
Molecular function GO term	GO:0005515;protein binding;<br>GO:0005524;ATP binding	GO:0005515;protein binding
Biological process GO term	GO:0006260;DNA replication	GO:0000074;regulation of progression through cell cycle;<br>GO:0006260;DNA replication
UniProt functional keyword matching	ATP-binding;<br>DNA replication;<br>DNA-binding;<br>Nuclear protein;<br>Nucleotide-binding;<br>Polymorphism;<br>Transcription;<br>Transcription regulation	
Cell-cycle stage	G1/S	
OMIM ID	602638	602696

Top 5	Protein A	Protein B
	ORC1_HUMAN	MCM6_HUMAN
Molecular function GO term	GO:0003677;DNA binding;<br>GO:0005515;protein binding	GO:0005524;ATP binding;<br>GO:0042802;identical protein binding
Biological process GO term	GO:0006270;DNA replication initiation	GO:0006260;DNA replication
UniProt functional keyword matching	ATP-binding;<br>DNA replication;<br>DNA-binding;<br>Nuclear protein;<br>Nucleotide-binding;<br>Polymorphism	
Cell-cycle stage	G1/S	
OMIM ID	601902	601806

Top 6	Protein A	Protein B
	PRI1_HUMAN	RFC4_HUMAN
Molecular function GO term	GO:0003896;DNA primase activity	GO:0005515;protein binding
Biological process GO term	GO:0006269;DNA replication, synthesis of RNA primer	GO:0006260;DNA replication;<br>GO:0006271;DNA strand elongation;<br>GO:0006281;DNA repair;<br>GO:0048015;phosphoinositide-mediated signaling
UniProt functional keyword matching	DNA replication;<br>Polymorphism	
Cell-cycle stage	S	
OMIM ID	176635	102577

Top 7	Protein A	Protein B
	CDC7_HUMAN	PRI1_HUMAN
Molecular function GO term	GO:0004674;protein serine/threonine kinase activity;<br>GO:0005515;protein binding	GO:0003896;DNA primase activity
Biological process GO term	GO:0000082;G1/S transition of mitotic cell cycle;<br>GO:0006270;DNA replication initiation;<br>GO:0007089;traversing start control point of mitotic c...;<br>GO:0008285;negative regulation of cell proliferation	GO:0006269;DNA replication, synthesis of RNA primer
UniProt functional keyword matching	Polymorphism;<br>Transferase	
Cell-cycle stage	S	
OMIM ID	603311	603078

Top 8	Protein A	Protein B
	RFC2_HUMAN	PRI1_HUMAN
Molecular function GO term	GO:0005515;protein binding;<br>GO:0005524;ATP binding	GO:0003896;DNA primase activity
Biological process GO term		GO:0006269;DNA replication, synthesis of RNA primer
UniProt functional keyword matching	DNA replication;<br>Polymorphism	
Cell-cycle stage	S	
OMIM ID	600404	176635

**Top 9**	**Protein A**	**Protein B**
	SMC4_HUMAN	KINH_HUMAN
Molecular function GO term	GO:0005524;ATP binding;<br>GO:0046982;protein heterodimerization activity	GO:0003777;microtubule motor activity
Biological process GO term	GO:0007001;chromosome organization and biogenesis;<br>GO:0007076;mitotic chromosome condensation	GO:0007018;microtubule-based movement
UniProt functional keyword matching	ATP-binding;<br>Coiled coil;<br>Nucleotide-binding	
Cell-cycle stage	G2	
OMIM ID	601902	601806

**Top 10**	**Protein A**	**Protein B**
	H2BQ_HUMAN	TOP1_HUMAN
Molecular function GO term	GO:0003677;DNA binding	GO:0003917;DNA topoisomerase type I activity
Biological process GO term	GO:0006334;nucleosome assembly	
UniProt functional keyword matching	DNA-binding	
Cell-cycle stage	M/G1	
OMIM ID	601831	126420

Top 11	Protein A	Protein B
	IMB1_HUMAN	H2BQ_HUMAN
Molecular function GO term	GO:0005515;protein binding; GO:0008139;nuclear localization sequence binding; GO:0008270;zinc ion binding	GO:0003677;DNA binding
Biological process GO term	GO:0000060;protein import into nucleus, translocation; GO:0006607;NLS-bearing substrate import into nucleus	GO:0006334;nucleosome assembly
UniProt functional keyword matching	Nuclear protein	
Cell-cycle stage	M/G1	
OMIM ID	602738	601831

Top 12	Protein A	Protein B
	PRI2_HUMAN	RFC4_HUMAN
Molecular function GO term	GO:0003896;DNA primase activity	GO:0005515;protein binding
Biological process GO term	GO:0006269;DNA replication, synthesis of RNA primer	GO:0006260;DNA replication; GO:0006271;DNA strand elongation; GO:0006281;DNA repair; GO:0048015;phosphoinositide-mediated signaling
UniProt functional keyword matching	DNA replication	
Cell-cycle stage	S	
OMIM ID	176636	102577

Top 13	Protein A	Protein B
	PRI2_HUMAN	RFC2_HUMAN
Molecular function GO term	GO:0003896;DNA primase activity	GO:0005515;protein binding; GO:0005524;ATP binding
Biological process GO term	GO:0006269;DNA replication, synthesis of RNA primer	
UniProt functional keyword matching	Alternative splicing; DNA replication	
Cell-cycle stage	S	
OMIM ID	176636	600404

Top 14	Protein A	Protein B
	PRI1_HUMAN	RFA2_HUMAN
Molecular function GO term	GO:0003896;DNA primase activity	GO:0003697;single-stranded DNA binding; GO:0005515;protein binding
Biological process GO term	GO:0006269;DNA replication, synthesis of RNA primer	GO:0006261;DNA-dependent DNA replication
UniProt functional keyword matching	DNA replication; Polymorphism	
Cell-cycle stage	S	
OMIM ID	176635	179836

Top 15	Protein A	Protein B
	HMGB2_HUMAN	H2AX_HUMAN
Molecular function GO term	GO:0003690;double-stranded DNA binding; GO:0003697;single-stranded DNA binding; GO:0003700;transcription factor activity; GO:0008301;DNA bending activity	GO:0003677;DNA binding; GO:0005515;protein binding
Biological process GO term	GO:0006260;DNA replication; GO:0006268;DNA unwinding during replication; GO:0006281;DNA repair; GO:0006288;base-excision repair, DNA ligation; GO:0006325;establishment and/or maintenance of chromat...; GO:0006334;nucleosome assembly; GO:0006357;regulation of transcription from RNA polyme...; GO:0048015;phosphoinositide-mediated signaling	GO:0007001;chromosome organization and biogenesis; GO:0006334;nucleosome assembly
UniProt functional keyword matching		
Cell-cycle stage	G2	
OMIM ID	163906	

Top 16	Protein A	Protein B
	H2AX_HUMAN	CHK2_HUMAN
Molecular function GO term	GO:0003677;DNA binding; GO:0005515;protein binding	GO:0005515;protein binding; GO:0004674;protein serine/threonine kinase activity
Biological process GO term	GO:0007001;chromosome organization and biogenesis; GO:0006334;nucleosome assembly	GO:0000077;DNA damage checkpoint; GO:0006974;response to DNA damage stimulus
UniProt functional keyword matching		
Cell-cycle stage	G2	
OMIM ID		604373

Top 17	Protein A	Protein B
	IMDH1_HUMAN	ERBB3_HUMAN
Molecular function GO term	GO:0003938;IMP dehydrogenase activity	GO:0005006;epidermal growth factor receptor activity;<br>GO:0005515;protein binding;<br>GO:0046982;protein heterodimerization activity
Biological process GO term		GO:0006468;protein amino acid phosphorylation;<br>GO:0007165;signal transduction
UniProt functional keyword matching	3D-structure;<br>Alternative splicing	
Cell-cycle stage		
OMIM ID	146690	190151

Top 18	Protein A	Protein B
	RASH_HUMAN	ERBB3_HUMAN
Molecular function GO term	GO:0008022;protein C-terminus binding	GO:0005006;epidermal growth factor receptor activity;<br>GO:0005515;protein binding;<br>GO:0046982;protein heterodimerization activity
Biological process GO term	GO:0000074;regulation of progression through cell cycle;<br>GO:0006935;chemotaxis;<br>GO:0007166;cell surface receptor linked signal transduction;<br>GO:0009887;organ morphogenesis	GO:0006468;protein amino acid phosphorylation;<br>GO:0007165;signal transduction
UniProt functional keyword matching	3D-structure;<br>Membrane;<br>Nucleotide-binding	
Cell-cycle stage		
OMIM ID	190020	190151

Top 19	Protein A	Protein B
	FRK_HUMAN	TEC_HUMAN
Molecular function GO term	GO:0004715;non-membrane spanning protein tyrosine kinase activity	GO:0004715;non-membrane spanning protein tyrosine kinase activity
Biological process GO term	GO:0000074;regulation of progression through cell cycle; GO:0006468;protein amino acid phosphorylation; GO:0008285;negative regulation of cell proliferation	GO:0006468;protein amino acid phosphorylation; GO:0007243;protein kinase cascade
UniProt functional keyword matching	ATP-binding; Kinase; Nucleotide-binding; Phosphorylation; SH2 domain; SH3 domain; Transferase; Tyrosine-protein kinase	
Cell-cycle stage		
OMIM ID	606573	600583

Top 20	Protein A	Protein B
	RHOC_HUMAN	ERBB3_HUMAN
Molecular function GO term	GO:0003924;GTPase activity; GO:0004871;signal transducer activity	GO:0005006;epidermal growth factor receptor activity; GO:0005515;protein binding; GO:0046982;protein heterodimerization activity
Biological process GO term	GO:0043123;positive regulation of I-kappaB kinase/NF-kappaB cascade	GO:0006468;protein amino acid phosphorylation; GO:0007165;signal transduction
UniProt functional keyword matching	3D-structure; Membrane; Nucleotide-binding	
Cell-cycle stage		
OMIM ID	165380	190151
